# Quantifying distribution shifts in single-cell data with scXMatch

**DOI:** 10.1093/bioinformatics/btag706

**Published:** 2026-10-03

**Authors:** Anna Möller, Miriam Schnitzerlein, Eric Greto, Vasily Zaburdaev, Stefan Uderhardt, David B Blumenthal

**Affiliations:** Biomedical Network Science Lab, Department of Artificial Intelligence in Biomedical Engineering, Friedrich-Alexander-Universität Erlangen-Nürnberg, Erlangen, 91052, Germany; Department of Medicine 3—Rheumatology and Immunology, Friedrich-Alexander-Universität Erlangen-Nürnberg and Universitätsklinikum Erlangen, Erlangen, 91054, Germany; Department of Biology, Friedrich-Alexander-Universität Erlangen-Nürnberg, Erlangen, 91058, Germany; Max-Planck-Zentrum für Physik und Medizin, Erlangen, 91058, Germany; Max Planck Institute for the Science of Light, Erlangen, 91058, Germany; Department of Medicine 3—Rheumatology and Immunology, Friedrich-Alexander-Universität Erlangen-Nürnberg and Universitätsklinikum Erlangen, Erlangen, 91054, Germany; Deutsches Zentrum für Immuntherapie, Friedrich-Alexander-Universität Erlangen-Nürnberg, Erlangen, 91054, Germany; Exploratory Research Unit, Optical Imaging Competence Center Erlangen, Friedrich-Alexander-Universität Erlangen-Nürnberg, Erlangen, 91058, Germany; Department of Biology, Friedrich-Alexander-Universität Erlangen-Nürnberg, Erlangen, 91058, Germany; Max-Planck-Zentrum für Physik und Medizin, Erlangen, 91058, Germany; Department of Medicine 3—Rheumatology and Immunology, Friedrich-Alexander-Universität Erlangen-Nürnberg and Universitätsklinikum Erlangen, Erlangen, 91054, Germany; Deutsches Zentrum für Immuntherapie, Friedrich-Alexander-Universität Erlangen-Nürnberg, Erlangen, 91054, Germany; Exploratory Research Unit, Optical Imaging Competence Center Erlangen, Friedrich-Alexander-Universität Erlangen-Nürnberg, Erlangen, 91058, Germany; Biomedical Network Science Lab, Department of Artificial Intelligence in Biomedical Engineering, Friedrich-Alexander-Universität Erlangen-Nürnberg, Erlangen, 91052, Germany

## Abstract

**Motivation:**

A basic task that frequently arises when analyzing single-cell data is to assess if there is a global distribution shift between the data profiles of cells from two different conditions. Widely used approaches to address this task such as visual inspection of two-dimensional representations or clustering-based workflows lack a solid statistical underpinning and are notoriously unstable and prone to confirmation bias.

**Results:**

To promote more rigorous analysis, we present scXMatch. scXMatch is based on a non-parametric graph-based test to quantify distribution shifts in arbitrary data spaces for which a suitable distance measure is available. We evaluated scXMatch on single-cell gene expression, chromatin accessibility, and imaging-derived cell morphology data, showing that it can robustly detect distribution shifts for different types of single-cell data. scXMatch thus aims to set a new standard in the single-cell biology field, replacing easy-to-manipulate semi-manual distribution shift quantification workflows by principled statistical testing.

**Availability and implementation:**

Python source code of scXMatch is available at https://github.com/bionetslab/scxmatch, a packaged version at https://anaconda.org/bioconda/scxmatch, and scripts to reproduce our results at https://github.com/bionetslab/scXMatch_paper (stable releases at https://doi.org/10.5281/zenodo.20665675).

## 1 Introduction

The following question arises in many studies in the field of single-cell biology: Is there a global distribution shift between profiles of cells of the same type in a target condition in comparison to a control condition (e.g. diseased versus healthy, treated versus untreated, knockout versus wild-type)? To the best of our knowledge, the only method specifically proposed for answering this question is the permutation-based *E*-distance test (Peidli *et al.* 2024), which relies on principal component analysis (PCA), subsampling, and computation of Euclidean distances in PCA space. Depending on the type of data—single-cell RNA sequencing (scRNA-seq), single-cell ATAC sequencing (scATAC-seq), etc.—other typical workflows include:

- Visually inspect the data upon projection into a two-dimensional (2D) space, e.g. PCA followed by popular dimensionality reduction techniques such as UMAP (McInnes et al. 2018) or t-SNE (Van der Maaten and Hinton 2008). Conclude that there is a global distribution shift if the 2D representations of the cells from the different conditions are visually separated.
- Carry out joint clustering of the single-cell data for the two conditions, followed by cluster-level over-representation analysis. Conclude that there is a global distribution shift if the cells from the different conditions are unevenly distributed over the clusters.
- Run statistical methods to identify individual differential features in the high-dimensional data, i. e., single variables such as differentially expressed genes (DEGs) or differentially accessible peaks that behave differently between the two conditions. Conclude that there is a global distribution shift if there are many differential features.
- Train a machine learning (ML) model that aims to separate the cells from the two conditions based on their data. Conclude that there is a global distribution shift if the ML model can accurately separate the two conditions.

Although widely used, all of these approaches have known shortcomings, which are particularly well studied for scRNA-seq data. For instance, 2D representations of scRNA-seq data have been shown to poorly preserve distances in the original high-dimensional data space, casting doubt on the reliability of visual inspection of 2D data representations (Chari and Pachter 2023). Clustering-based workflows are problematic because clustering scRNA-seq and other single-cell data is notoriously sensitive to the choice of the clustering algorithm (Yu *et al.* 2022, Liang *et al.* 2023). Similar problems apply to workflows that rely on differential feature counts or ML-based separability as also here, different subroutines (i. e., different ML models or different methods to identify differential features) often produce substantially different results (Soneson and Robinson 2018, Squair *et al.* 2021). Moreover, differential feature counts are often fragile and can be massively inflated when there are no or too few biological replicates and cells are treated as independent observations during differential testing (Squair *et al.* 2021). More generally, all of these approaches are indirect, at least to some degree *ad hoc*, and easily lead to confirmation bias: Many hyper-parameters and subroutines have to be selected, and it is almost always close to impossible to make principled and non-arbitrary decisions regarding all of these steps. Given enough trial and error, it is hence very often possible to achieve the “desired” results.

To address these problems, we here present scXMatch—a Python package to directly test for global distribution shifts between sets $X_{1}=\{x_{i}|y_{i}=1\}$ and $X_{0}=\{x_{i}|y_{i}=0\}$ of *h*-dimensional profiles $x_{i}\in R^{h}$ of cells *i* from a target condition $y_{i}=1$ or a control condition $y_{i}=0$. scXMatch implements a runtime- and memory-efficient version of the cross-matching test proposed by Rosenbaum (2005). The cross-matching test is a non-parametric approach to test the null hypothesis that two sets of multivariate data points $X_{0}$ and $X_{1}$ were sampled from the same distribution $F_{0}=F_{1}$. It is ideally suited to assess global distribution shifts in single-cell data, as it relies on a test statistic whose distribution under the null hypothesis can be computed exactly even when the data-generating distribution $F_{0}=F_{1}$ is unknown (more details in Section 2 and Online Appendix A). The only required input besides $X_{0}$ and $X_{1}$ is a suitable distance measure $d:R^{h}\times R^{h}\to R_{\geq 0}$ to quantify cell profile dissimilarity.

Despite these desirable properties and although there is an R implementation of the cross-matching test (Heller *et al.* 2024, https://CRAN.R-project.org/package=crossmatch), we are not aware of a single study which uses it to assess global distribution shifts in single-cell omics data such as scRNA-seq or scATAC-seq data. One likely reason for this is that the original version requires to compute a minimum-weight maximum-cardinality matching (MWMCM) in a complete weighted graph, where each data profile $x_{i}$ is represented by a node. This requires quadratic memory with respect to the number of data profiles, which makes out-of-the-box usage of the cross-matching test infeasible for large-scale single-cell datasets. For scXMatch, we therefore developed a substantially more efficient version of the cross-matching test, where the complete graph is replaced by a sparse *k*-nearest neighbor (*k*-NN) graph, which reduces the test’s memory requirements from quadratic to linear. A systematic evaluation on scRNA-seq, scATAC-seq, and imaging-derived morphodynamics data shows that scXMatch scales to realistically sized single-cell datasets and that it can reliably distinguish signal from noise, using experimental perturbation of varying strength as ground truth. A theoretical investigation shows that, for the special case of 1D data, scXMatch is strictly more sensitive than the Wilcoxon rank-sum test. To further encourage adoption within the single-cell biology research community, we implemented scXMatch as an scverse-compatible Python package and published it in the scverse ecosystem (Virshup *et al.* 2023).

## 2 Method overview

The general purpose of scXMatch is to examine whether two groups $X_{0}$ and $X_{1}$ of single-cell data profiles are sampled from the same distribution. Figure 1 provides a high-level overview of scXMatch; see Online Appendix A and the original publication of the cross-matching test (Rosenbaum 2005) for further details.

**Figure 1:**
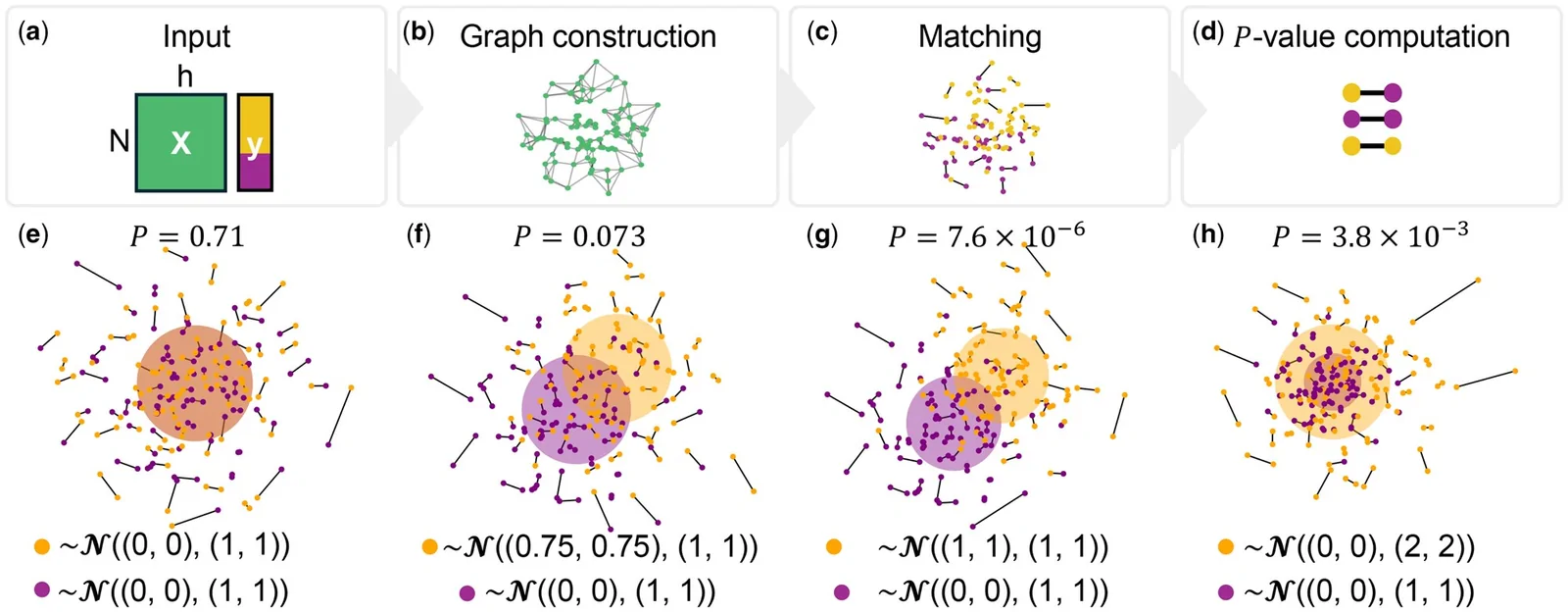
Overview of scXMatch. (a) Required inputs: *N* single-cell profiles $x_{i}$, binary condition labels $y_{i}$ (orange/purple), a dissimilarity measure *d*, and the *k* parameter for *k*-NN mode. (b–c) A weighted graph *G* (complete or *k*-NN) is constructed, and a minimum-weight matching *M* is computed. (d) The number of cross-matches between conditions yields a *P*-value to assess global distribution shifts. (e–h) Examples using synthetic data sampled from bivariate normal distributions show that scXMatch detects significant shifts when distributions differ in mean (f, g) or variance (h), but not when they are identical (e).

First, scXMatch constructs a weighted undirected connectivity graph *G*, where the node set corresponds to the set of all data profiles $X=X_{0}\cup X_{1}$ and edges connecting two cells *i* and *j* are weighted by the dissimilarity $d(x_{i},x_{j})$ of their profiles. Depending on available computational resources and the size of X, *G* can be constructed as the complete graph or as a *k*-NN graph. Next, scXMatch computes a MWMCM *M* in *G*, meaning that as many cells as possible are assigned to exclusive pairs, such that the sum of distances between the features of paired cells is minimized.

The pairs ij∈M are either iso-matches or cross-matches, depending on whether the paired cells *i* and *j* are from the same condition ($y_{i}=y_{j}$) or from different conditions ($y_{i}\neq y_{j}$). The number of cross-matches $a_{1}$ is used as a test statistic, and a *P*-value, a *z*-score, and a sample size-independent effect strength ratio $R=1-\text{min}\{a_{1},E(A_{1})\}/E(A_{1})$ are computed based on the distribution of the corresponding random variable $A_{1}$. Under the null hypothesis that $X_{0}$ and $X_{1}$ are sampled from the same distribution $F_{0}=F_{1}$, the distribution of $A_{1}$ is exactly known even if $F_{0}=F_{1}$ is unknown (as shown in Online Appendix A, this also holds for our efficient *k*-NN graph-based variant of the cross-matching test). The smaller the number of cross-matches $a_{1}$, the smaller the likelihood of observing it under the null hypothesis. A small number of cross-matches is thus indicative of a distribution shift between $X_{0}$ and $X_{1}$. A major advantage of scXMatch is that it is very flexible regarding its input. Although designed with single-cell data in mind, it can be used for any kind of multivariate data, as long as a suitable dissimilarity measure *d* is available. We provide out-of-the-box support for all distance functions implemented in SciPy; custom dissimilarity measures can be provided by the user.

## 3 Experimental results

### 3.1 scXMatch scales to large datasets

Besides the dissimilarity measure *d*, scXMatch requires the number of neighbors *k* as an additional hyperparameter when run in *k*-NN mode. In principle, it makes sense to select *k* as large as possible, since smaller *k* more strongly restrict the solution space for the MWMCM *M* (see Online Appendix A for details). However, setting *k* to larger values increases the runtime and memory requirements of scXMatch. To provide a guideline on how to select *k*, we therefore simulated *N* data points $x_{i}\in R^{h}$ (corresponding to data profiles of individual cells in the context of single-cell data) for all combinations (N,h)∈{500,1000,2000,5000,10000,20000,50000}×{10,500,1000,2000}, using both Gaussian distributions and Splatter (Zappia *et al.* 2017)—a tool specifically designed for simulating scRNA-seq data. On each of the resulting 56 simulated datasets, we ran scXMatch with squared Euclidean distance [as recommended for scRNA-seq data (Ji *et al.* 2023)] and with varying k∈{10,20,50,100,200,500,1000,2000,5000} and recorded the peak memory usage, the overall runtime, and the relative coverage *cov* of the computed MWMCM *M*, i. e. the fraction of data profiles used towards the final *P*-value computation. Each run was executed on 16 CPU cores with sufficient memory resources. Figure 2 shows the results. Overall, we identified main memory usage as the major limiting factor. To enable the user to approximate the expected memory requirements, we fitted a linear regression model that estimates the total RAM usage based on the number of edges |*E*| in the *k*-NN graph *G*, using data from all scXMatch runs on simulated Gaussian and Splatter data. Since the obtained model


(1) $$\text{estimated}\;\text{total}\;\text{RAM}[\text{GB}]\;=\;\beta_{0}+\beta_{1}|E|$$

**Figure 2:**
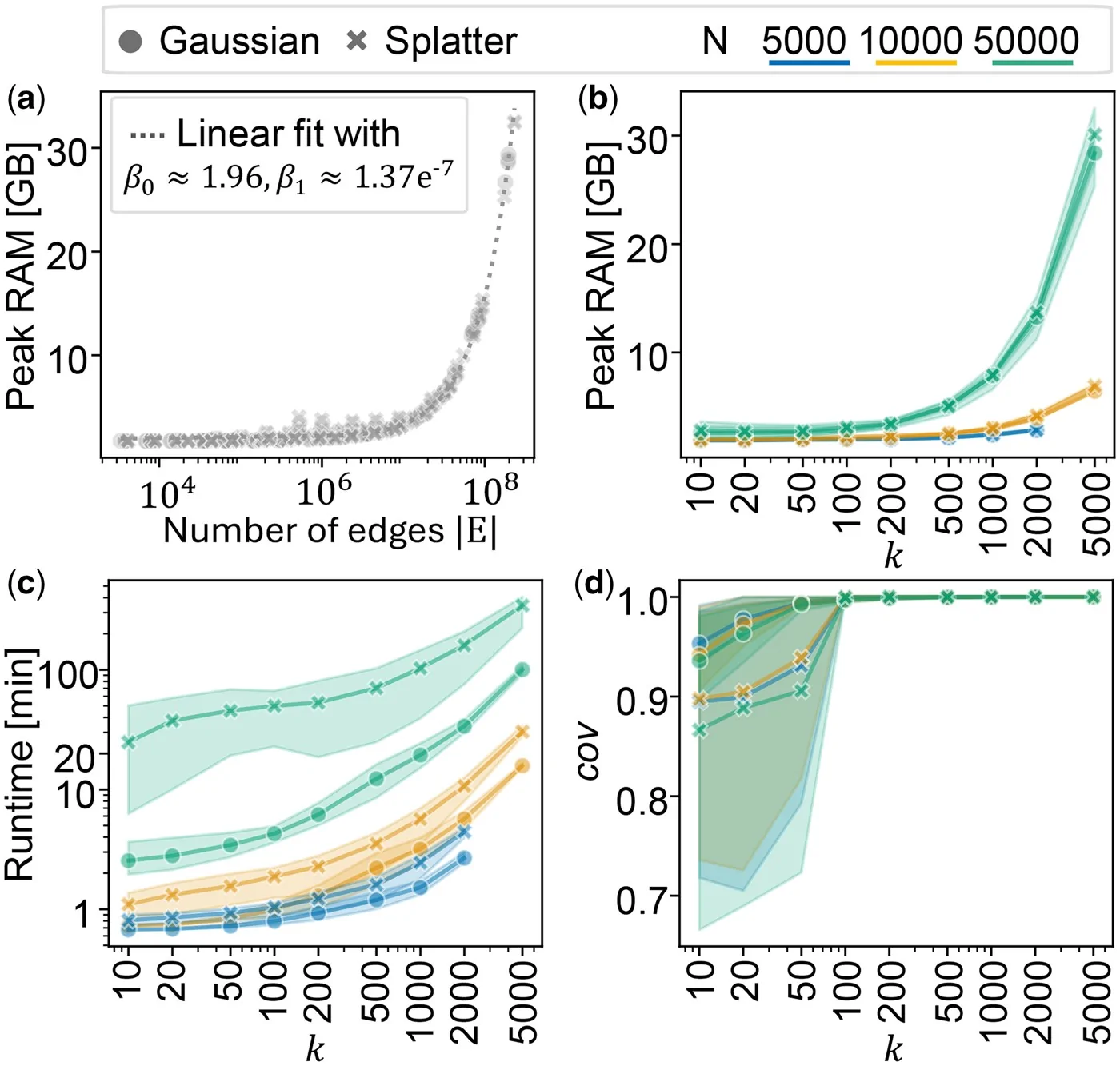
Results of the scalability tests. (a) Peak memory usage as a function of the number of edges |*E*| in scXMatch’s *k*-NN graph. (b–d) Peak memory usage (b), runtime (c), and relative coverage (d) as a function of *k*, for datasets with different numbers of samples *N*. Line plots and shaded areas in (b–d) depict means and 95% confidence intervals obtained for datasets with fixed *N* and *k* and varying dimensionality h∈{10,500,1000,2000}.

(with $\beta_{0}\approx 1.96$ and $\beta_{1}\approx 1.37e^{-7}$) excellently fits the data (Fig. 2a) and |*E*| is bounded from above by |E|≤Nk (how closely |*E*| approaches this bound depends on the symmetry of the *k*-NN relationships), we implemented an estimate_peak_RAM_GB() function in scXMatch’s Python package. The function allows the user to estimate the worst-case RAM requirements as $\beta_{0}+\beta_{1}Nk$ prior to running the test and to thus select a suitable value of *k*, given the size of their data and their available resources.

Figure 2 also contains overviews on peak memory usage (Fig. 2b) and runtime (Fig. 2c) as a function of *k* for different *N*. The dimensionality *h* of the input data only has a very small effect on scXMatch’s memory requirements, as can be seen by the tight error margins of the line plots in Fig. 2b. The test finished in under a minute for any value of *k* for datasets with N=2000 samples, and in under 18 minutes with k≤1000 for the random Gaussian datasets with N=50000 samples. For the extremely sparse Splatter data, running scXMatch takes 10 to 70 minutes on N=50000 samples, depending on the dimensionality. With k≥100, the relative coverage—which indicates how many samples were covered by the matching—exceeds 99% for both Gaussian and Splatter data and all combinations of *N* and *h* (Fig. 2d). For the experiments on scRNA-seq data presented below, we thus used k=100, as this setup runs in reasonable time and memory and covers a convincing amount of samples (Table C.1 in Online Appendix C). A small ablation study moreover showed that the *P*-values are very robust w. r. t. the choice of *k* when run on scRNA-seq data (Fig. C.1 in Online Appendix C). The scATAC-seq and morphodynamics datasets were smaller, which allowed us to run scXMatch in full graph mode.

**Table 1:** Benjamini-Hochberg corrected *P*-values obtained when running scXMatch and the *E*-distance test on scATAC-seq data of human control T cells and T cells subjected to targeted CRISPR-Cas9 knockout (KO) of different genes, sorted by decreasing scXMatch significance.

KO genes	Adj. *P* (scXMatch)	Adj. *P* (*E*-distance)
**2000 most variable genes**		
CD3E	1.2×10${\;}^{-25}$	6×10${\;}^{-4}$
CD3E + CD4	1.7×10${\;}^{-20}$	6×10${\;}^{-4}$
ZAP70	3.5×10${\;}^{-11}$	6×10${\;}^{-4}$
NFKB2	0.58	6×10${\;}^{-4}$
CD4	1.0	6×10${\;}^{-4}$
**TNF-** α **signaling via NF-**κ**B pathway genes**		
CD3E	2.6×10${\;}^{-38}$	6×10${\;}^{-4}$
CD3E + CD4	4.5×10${\;}^{-39}$	6×10${\;}^{-4}$
ZAP70	2.9×10${\;}^{-18}$	6×10${\;}^{-4}$
NFKB2	8.1×10${\;}^{-5}$	6×10${\;}^{-4}$
CD4	0.31	6×10${\;}^{-4}$

Top: Results when running the two tests on the 2000 most variable genes. Bottom: Results when restricting the input to genes from the MSigDB pathway “TNF-α signaling via NF-κB.” The *E*-distance test was run in four configurations ($K\in \{10^{4},2\times 10^{4}\}$ permutations, with and without subsampling), which all yielded the same *P*-values reported in this table.

### 3.2 scXMatch reliably detects global distribution shifts in scRNA-seq data

A method designed to quantify global distribution shifts in single-cell data should yield scores that reflect the strength of underlying biological differences. To test if this is the case, we used ten scRNA-seq datasets from studies where cells were exposed to perturbations of increasing strengths (Fig. 3a): The datasets contain scRNA-seq data for cells of different cell lines at different time points after drug treatment (McFarland *et al.* 2020), for K562 cells upon single- and dual-guide CRISPR overexpression (Norman *et al.* 2019), for embryonic fibroblasts at different time points after transcription factor-mediated cellular reprogramming (Schiebinger *et al.* 2019), and for different neuron subtypes in the prefrontal cortex of mice that were withdrawn for 48 hours or 15 days after cocaine addiction (Bhattacherjee *et al.* 2019). We ran scXMatch with k=100 and the squared Euclidean distance as dissimilarity measure *d*, following the recommendation of a benchmark on distance measures for scRNA-seq data (Ji *et al.* 2023). As a competitor, we included the pertpy (Heumos *et al.* 2026) implementation of the *E*-distance test (Peidli *et al.* 2024) in the default configuration suggested by the authors (subsampling to the size of the smallest cell population, Euclidean distance computation based on the first 50 principal components, $K=10^{4}$ permutations), as well as three additional configurations ($K=10^{4}$ without subsampling, $K=2\times 10^{4}$ with subsampling, $K=2\times 10^{4}$ without subsampling).

**Figure 3:**
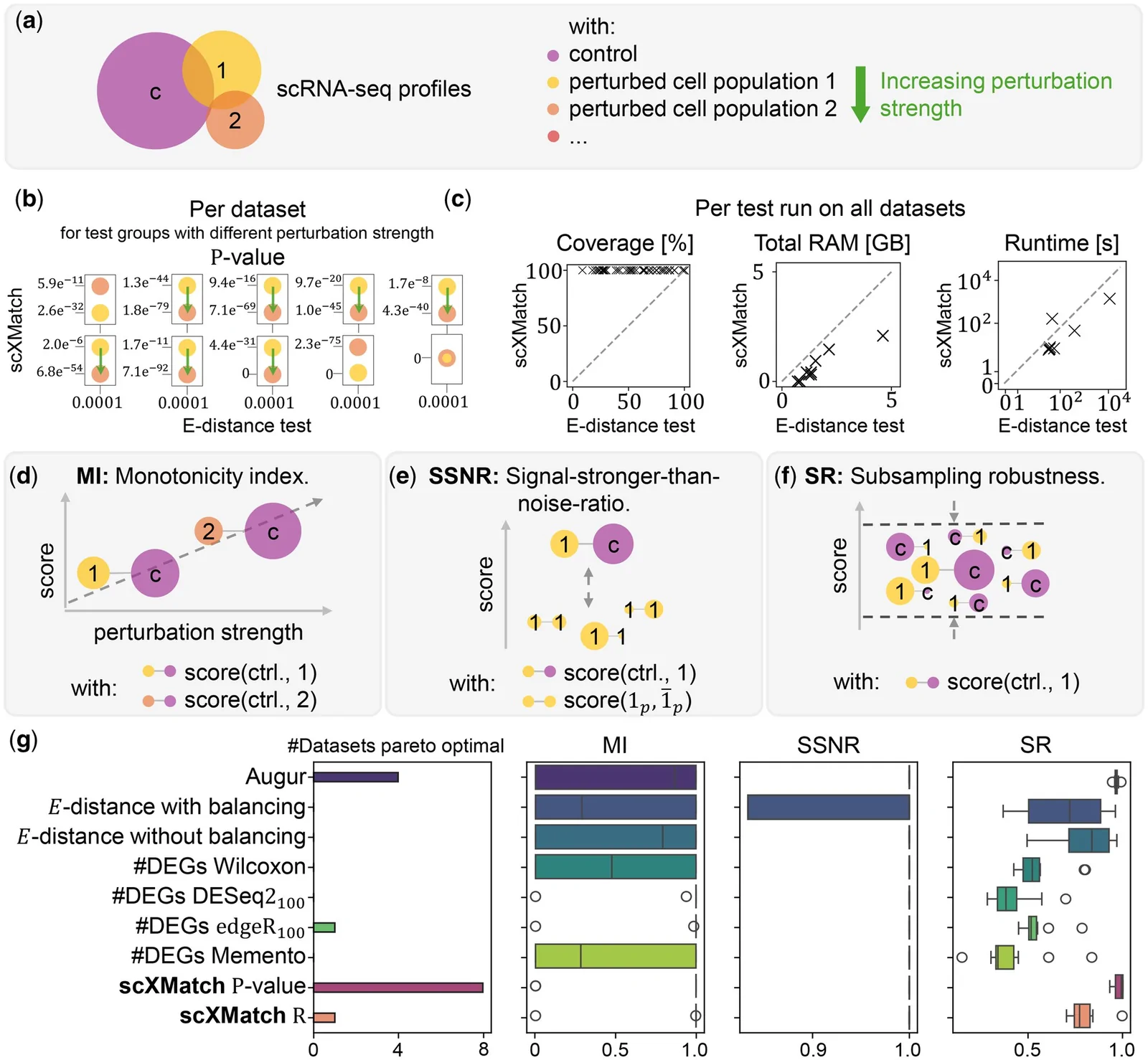
Benchmarking results on scRNA-seq data. (a) We used ten public scRNA-seq datasets from cells that were subject to perturbations of increasing strengths. (b) *P*-values obtained with the *E*-distance test (*x*-axes, results are identical for all four tested configurations) and scXMatch (*y*-axes) when comparing control against perturbed cell populations on the ten datasets (columns). Downwards arrows indicate alignment of *P*-values with perturbation strength. (c) Direct comparison of the relative coverages, required RAM, and runtimes of scXMatch and the *E*-distance test run in the default configuration suggested by the authors. (d–g) For comparison of our tool scXMatch against commonly used approaches that assess distribution shifts in scRNA-seq data we defined three metrics. (d) MI: Comparing cell populations with increasing perturbation strength against an unperturbed reference population should lead to increasing scores. (e) SSNR: Comparing a perturbed population against the reference should lead to a higher score than comparing disjoint subsets of the perturbed population against each other. (f) SR: Comparing different subsets of the reference against different subsets of the same perturbed condition should lead to similar scores as the comparison of the full data. (g) Benchmarking results of the nine approaches on all ten datasets. For eight of the datasets, scXMatch’s *P*-value is Pareto-optimal with regard to the three metrics (left panel).

For all comparisons of control against perturbed cell populations, the *E*-distance test returned the smallest possible empirical *P*-value for all four tested configurations, indicating that it is very sensitive to distribution shifts but cannot distinguish between distribution shifts of different strengths (Fig. 3b, *x*-axes). In contrast, scXMatch returned *P*-values whose differences align with the biological expectations in eight out of ten datasets (Fig. 3b, *y*-axes). scXMatch also clearly outperformed the subsampling-based default configuration of the *E*-distance test in terms of relative coverage, showing that, unlike the *E*-distance test’s subsampling approach, scXMatch’s strategy to compute MWMCMs in a *k*-NN graph retains almost all cells for the final *P*-value computation (Fig. 3c, left panel). An experiment where we subsampled two perturbed cell populations from one benchmark dataset showed that this statement remains true when using scXMatch in strongly imbalanced scenarios where one of the two compared cell populations is tiny (Fig. C.2 in Online Appendix C). Moreover, scXMatch is substantially more memory- and runtime-efficient than the *E*-distance test: On the largest benchmark dataset, scXMatch was around 17.33 times faster and required 2.23 times less RAM than the *E*-distance test’s default configuration (Fig. 3c, middle and right panels); when comparing against configurations without subsampling or a larger number of permutations, these factors increase substantially (Fig. C.3 in Online Appendix C).

We also benchmarked scXMatch’s *P*-values against other typical workflows that are often used to quantify global distribution shifts. As very simple baseline, we included the number of DEGs between the two compared cell populations, with DEGs determined using edgeR (Robinson *et al.* 2010), DESeq2 (Love *et al.* 2014), the Wilcoxon rank-sum test (Wilcoxon 1992), and Memento (Kim *et al.* 2024b), respectively. Since edgeR and DESeq2 require bulk input, we employed a pseudo-bulk strategy by randomly grouping cells within each condition and biological replicate (if available) in batches of size 100 (pseudo-bulking by biological replicates alone as suggested by Squair *et al.* (2021) was impossible due to insufficient availability of replicates in our datasets). Moreover, we tested against the Augur score—a more advanced competitor approach which quantifies global distribution shifts as ML-based separability (Skinnider *et al.* 2021). While Augur is typically used to assess cell-type-specific responsiveness to changing conditions, we here applied it in a global setting by evaluating its ability to discriminate between conditions in a single cell type. Finally, we included scXMatch’s effect strength ratio *R* and the raw *E*-distance (with and without subsampling) into the benchmark. *E*-distance *P*-values were not included in the extended benchmark because they were identical for comparisons between control and perturbed cell populations, as discussed above and shown in Fig. 3b.

We defined three quantitative evaluation metrics to compare scXMatch against these competitor workflows (see Fig. 3d–f for a visual explanation and Online Appendix A for formal definitions):

-
MI(s) (Fig. 3d): The monotonicity index MI(s)∈[0,1] measures if a distribution shift score *s* increases monotonically with increasing perturbation strength. Larger is better. Perturbation strength is defined separately for each dataset based on the experimental design (details in Online Appendix A).
-
SSNR(s) (Fig. 3e): The signal-stronger-than-noise ratio SSNR(s)∈[0,1] compares distribution shift scores from comparisons of condition against reference (signal) to scores from comparisons within conditions (noise). Larger is better.
-
SR(s) (Fig. 3f): The subsample robustness SR(s)≤1 quantifies robustness by evaluating the relative error of a distribution shift score across different subsampled versions of the same dataset, using the original dataset as reference. Larger is better and indicates that *s* remains stable under subsampling from the compared distributions.

The results of our benchmark are visualized in Fig. 3g. On eight of the ten datasets, scXMatch’s *P*-values are Pareto-optimal with regard to the three metrics. That is, scXMatch performs at least as good or better than all competitors w. r. t. all three metrics. In terms of monotonicity, scXMatch achieved a perfect MI of 1.0 for eight out of ten datasets. Regarding SSNR, all tools achieved perfect scores. With regard to SR, scXMatch’s *P*-values and Augur scores performed best, showing near-perfect robustness to dataset subsampling on all datasets, followed by the *E*-distances and scXMatch’s effect strength ratio *R*. The DEG-based scores obtained much lower SR scores than all competitors. This is an expected result: Since the small numbers of biological replicates in the scRNA-seq datasets precluded us from properly modeling replicate structure prior to DEG calling (see explanations above and in Online Appendix A), subsampling led to artificial variation in statistical power and, thus, in the number of called DEGs.

Finally, we assessed the false positive rate (FPR) of scXMatch’s *P*-values (for the competitor methods, we could not compute FPRs because they do not yield *P*-values): Out of all the 156 runs where we tested subsets of the same cell population against each other, scXMatch returned a significant raw *P*-value (P<0.05) in 2 cases, leading to FPR≈1.3% for raw *P*-values and FPR=0% after adjusting for multiple testing within each test group using Benjamini-Hochberg correction.

### 3.3 scXMatch implicitly removes outliers

Running scXMatch in *k*-NN mode can lead to a relative coverage below 100%—particularly in datasets with a large number of samples. This observation raises the question if samples that are not included in the final matching share certain characteristics. Since scXMatch constructs the matching based on a *k*-NN graph, samples that do not reside in densely connected regions of the distance graph are more likely to be excluded from the matching. These samples may correspond to outliers.

To systematically investigate this hypothesis, we computed local outlier factors (LOFs) (Breunig *et al.* 2000) for each cell based on the full pairwise distance matrix, using the Norman dataset as test case, where scXMatch run in *k*-NN mode with k∈{10,50,100} resulted in matching coverages below 100%. The LOF is a widely used density-based outlier score with LOF≈1 for inliers and LOF>1 for outliers. We then compared the distribution of LOFs between matched and unmatched cells, separately for each test group matched to its respective reference. Specifically, we performed one-sided Wilcoxon rank-sum tests with alternative hypothesis that the unmatched cells have larger LOFs than those included in the matching.

Figure 4 visualizes the LOF distributions, colored by whether or not a cell was included in the matching. Across all *k* and test groups, the alternative hypothesis was supported with high statistical significance. These findings suggest that scXMatch, when run in *k*-NN mode, effectively performs an implicit outlier removal step by excluding cells that are not embedded within well-connected regions of the data manifold.

**Figure 4:**
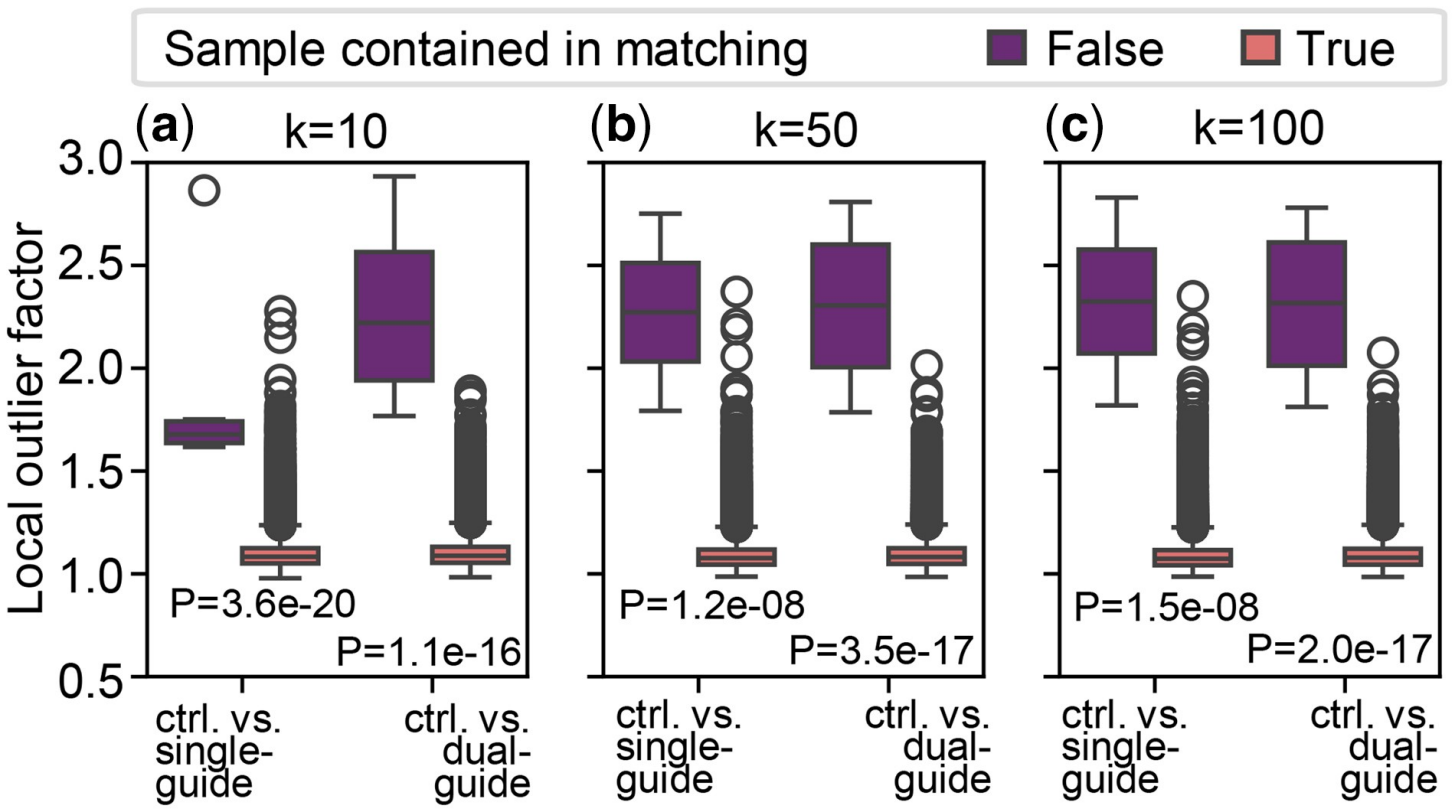
Local outlier factors, colored by inclusion in the MWMCMs obtained for the Norman dataset when using scXMatch with k∈{10,50,100} to compare perturbed cell populations to controls. Plots are annotated with *P*-values of the Wilcoxon rank-sum tests.

### 3.4 Changes in chromatin accessibility in scATAC-seq data identified by scXMatch reflect known T cell biology

scXMatch’s utility extends to other single-cell omics modalities beyond scRNA-seq data. To demonstrate this, we analyzed scATAC-seq data from Mimitou *et al.* (2021), which maps open chromatin regions primed for gene activation. CRISPR–Cas9 perturbations of signaling components of the T cell receptor (TCR) in human CD4+ T cells revealed context-dependent chromatin dynamics and accessibility. Due to the small sample sizes, we ran scXMatch in full graph mode (using the squared Euclidean distance as for scRNA-seq data) and could moreover include the resource-intensive *E*-distance test as a direct competitor.

Table 1 (upper panel) shows that scXMatch effectively ranked perturbation effects, with CD3E and ZAP70 knockouts yielding the most significant *P*-values. These upstream regulators are critical for structural TCR assembly (CD3E) and signal initiation/propagation (ZAP70), respectively, and drive chromatin remodeling by activating the transcription factors NFAT, AP-1, and NF-κB, which prime enhancers of activation-associated genes. Their disruption caused a global loss of chromatin accessibility, reflecting their non-redundant roles in early TCR signaling (Shah *et al.* 2021). In contrast, NFKB2 deficiency showed no significant effect, consistent with its role in sustaining late-phase responses rather than initiating chromatin remodeling (Sun 2011). Similarly, CD4 knockout had only a minimal effect on chromatin accessibility, since the anti-CD3/CD28 activation model used in this study bypasses the need for CD4’s typical function in MHC II recognition (Damle and Doyle 1989). scXMatch hence consistently prioritizes biologically coherent hits (more significant *P*-values for upstream vs. downstream effectors), underscoring its value for identifying global chromatin accessibility shifts. The *E*-distance test *P*-values did not relate to the importance of these different regulators, as, for all knockout groups, they were again all the minimal possible value. Running the *E*-distance tests with more permutations and/or without subsampling did not remedy this.

In addition to the quantification of global distribution shifts, scXMatch can also be used for pathway-level analyses. To exemplify this, we retrieved genes from the Molecular Signatures Database (MSigDB) hallmark gene set “TNF-α signaling via NF-κB” (Liberzon *et al.* 2015), and then used the accessibility scores of these genes as input for scXMatch and the *E*-distance test. We selected this pathway because we expected knockout of NFKB2 to more strongly alter chromatin accessibility within genes of this pathway than knockout of CD4, since NFKB2 encodes a core component of the NF-κB transcriptional regulatory machinery (Sun 2017), whereas CD4 functions primarily as an upstream T-cell co-receptor that modulates T-cell receptor signaling and can thus only indirectly influence NF-κB activation (Paul and Schaefer 2013). scXMatch’s *P*-values perfectly reflect this biologically informed expectation: for NFKB2 knockout, the *P*-value switches from non-significant to significant when running scXMatch on pathway genes only; for CD4 knockout, it remains non-significant. In contrast, the *E*-distance test again returned the smallest possible *P*-value for all comparisons (Table 1, lower panel).

### 3.5 scXMatch highlights importance of dynamic imaging for differentiating resident tissue macrophage populations

To exemplify the versatility of scXMatch beyond sequencing data, we used it to re-analyze data from our previous study on the morphology of differently stimulated resident tissue macrophages (RTMs) imaged *in vivo* in the peritoneum of mice (Schnitzerlein *et al.* 2025). RTMs are characterized by their dynamic changes in cell shape, as they perform constant sampling of the environment (Uderhardt *et al.* 2019). To describe those morphological characteristics, our previous study introduced a collection of 31 human-interpretable morphological RTM features, including 8 features that describe static RTM morphology and 23 features that quantify dynamic activity and adaptation to stimuli (Fig. 5a). Overall, the resulting dataset contains data for around 350 individual RTMs in control mice and upon stimulation with 5 different stimuli: the anti-inflammatory stimulants macrophage colony stimulating factor (MCSF) and transforming growth factor β (TGF-β), the pro-inflammatory stimulants lipopolysaccharides (LPS) and interferon γ (IFN-γ), and the kinase inhibitor YM201636 (YM), which induces a halt of the sampling activity.

**Figure 5:**
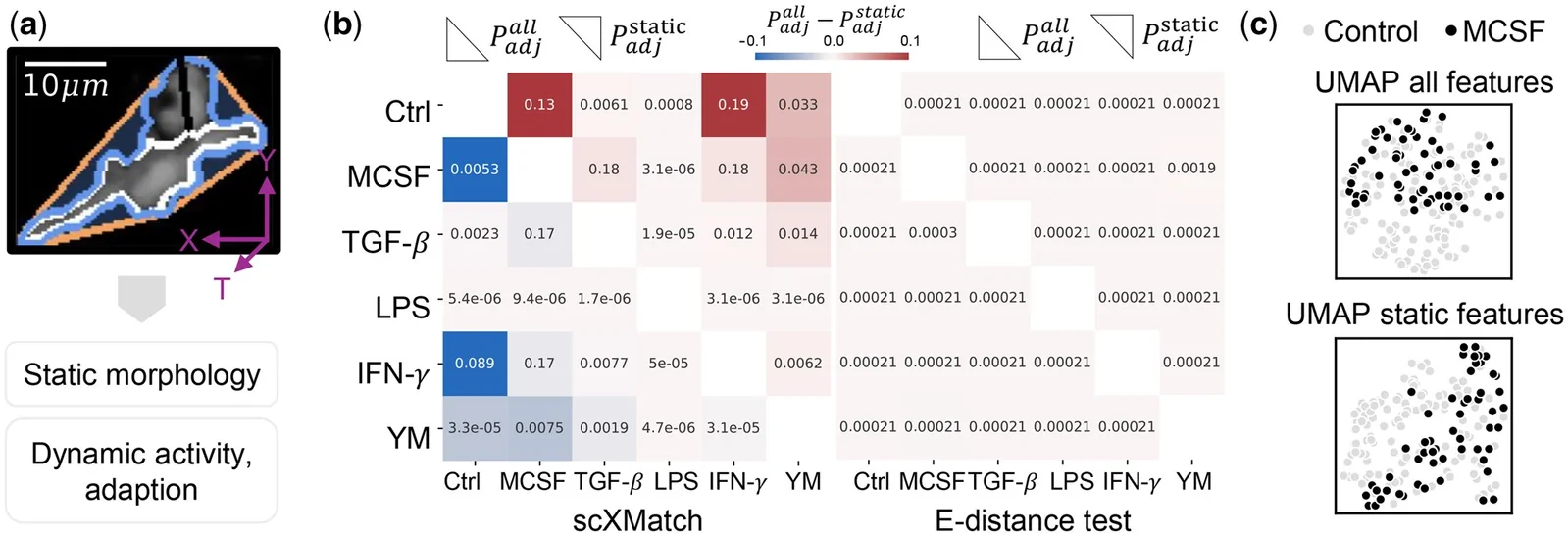
Importance of dynamic imaging data for distinguishing RTM populations. (a) Snapshot of an unperturbed, sampling macrophage *in vivo* from the study by Schnitzerlein *et al.* (2025). Several morphological single-cell features (as schematically drawn on the image) were extracted. They characterize three categories of properties: static morphology, dynamic activity, or adaptation capabilities. While the static features can be calculated from a single image, the latter two require time-lapse images. (b) Benjamini-Hochberg-adjusted *P*-values returned by scXMatch (left panel) and the *E*-distance test (right panel) for pairwise comparisons of RTM populations under different stimuli. Lower left triangle: adjusted *P*-values $P_{\mathrm{adj}}^{\mathrm{all}}$ obtained when using all features. Upper right triangle: adjusted *P*-values $P_{\mathrm{adj}}^{\mathrm{static}}$ obtained when using static features only. The heatmaps are colored according to the difference of these two values (lower triangle minus upper triangle). Strong colors indicate large differences. Since the RTM dataset is small, we could run scXMatch in full graph mode. The standardized Euclidean distance was used as a distance measure. (c) UMAPs of the control versus MCSF-treated RTMs calculated on all and on only the static features, respectively.

While our previous study identified distribution shifts in the 31-dimensional RTM morphology profiles between the different stimuli, we now sought to answer the question if the 8 static morphology quantifiers alone are sufficient to reliably distinguish RTM populations in different activation states. To do so, we used scXMatch and the *E*-distance test to carry out pairwise comparisons between the different RTM populations, using all features and the static features only, respectively. The results for scXMatch are shown in the left panel of Fig. 5b. Overall, we observe that differences become less pronounced when relying on static features only, highlighting the superiority of time-lapse *in vivo* imaging in capturing morphological RTM states over conventional imaging approaches that rely on fixated tissue samples (Zanier *et al.* 2015, Kim *et al.* 2024a). When assessing distribution shifts with the *E*-distance test, we encountered the issue that the recommended subsampling to equal group sizes led to poor relative coverage due to high group imbalance. Furthermore, results calculated on the balanced data strongly varied for repeated runs (see Fig. C.4 in Online Appendix C). When running the *E*-distance test without subsampling, we obtained more stable *P*-values, which however are all very similar and thus do not support any conclusions on the information content of dynamic features (Fig. 5b, right panel).

The strongest loss of significance in the scXMatch results can be observed for the comparison between MCSF-stimulated and control RTMs, where adjusted scXMatch *P*-values increase from 0.0053 when using all 31 morphological features (strongly significant) to 0.13 when using only the 8 static features (not significant). Figure 5c shows two-dimensional UMAP projections of the corresponding 31- and 8-dimensional data. While both projections suggest that the MCSF-stimulated RTMs span a subspace of the control RTMs, it is impossible to visually assess that one distribution shift is significant while the other one is not. This exemplifies the advantages of quantifying magnitudes of global distribution shifts with scXMatch in high-dimensional data instead of relying on subjective visual inspection of two-dimensional projections and shows that the additional effort of *in vivo* imaging is necessary to capture statistically significant changes in RTM morphology upon MCSF stimulation.

## 4 A link between scXMatch and the Wilcoxon rank-sum test

For the special case of 1D numeric data, we establish a theoretical link between scXMatch and the Wilcoxon rank-sum test. Although we developed scXMatch for high-dimensional rather than for 1D data, we believe that this link helps readers to gain a more thorough understanding of scXMatch’s mathematical foundations.

Recall that, for 1D numeric data, scXMatch’s test statistic $a_{1}$ is defined as the number of cross-matches contained in an MWMCM *M* of a connectivity graph *G* defined over the elements of $X=X_{0}\cup X_{1}\subset R$ with edge weights $w_{ij}=d(x_{i},x_{j})$. For our theoretical investigation, we assume the natural choice of $d(x_{i},x_{j})$ as the absolute difference $d(x_{i},x_{j})=|x_{i}-x_{j}|$. Moreover, we assume that X does not contain repeated elements (note that, while our proofs rely on this assumption, we conjecture that it may be possible to drop it). Under these assumptions, we show the following Theorem 1 (proof in Online Appendix B), which states that scXMatch’s test statistic $a_{1}$ can be upper-bounded by the test statistic of the two-sided Wilcoxon rank-sum test:

Theorem 1. *Assume that* $X=X_{0}\cup X_{1}\subset R$  *(*$X_{0}$  *sampled from* $F_{0}$, $X_{1}$  *sampled from* $F_{1}$*) and that* X  *does not contain repeated elements. Let the distance measure d be defined as* $d(x,x^{\prime })=|x-x^{\prime }|$*, let* $n=|X_{0}|$  *and* $m=|X_{1}|$  *be the numbers of elements in* $X_{0}$  *and* $X_{1}$*, and let* $U=\text{min}\{U_{0},U_{1}\}$  *with* $U_{0}=|\{(x,x^{\prime })\in X_{0}\times X_{1}|x<x^{\prime }\}|$  *and* $U_{1}=|\{(x,x^{\prime })\in X_{0}\times X_{1}|x>x^{\prime }\}|=nm-U_{0}$  *be the test statistic of the two-sided Wilcoxon rank-sum test with alternative hypothesis* $F_{0}\neq F_{1}$*. Moreover, let* $a_{1}$  *be the test statistic computed by scXMatch, run in full graph mode or in k-NN mode with* k≥2*. Then it holds that* $a_{1}\leq U+2$.

Since the *P*-values of both the cross-matching test implemented in scXMatch and the two-sided Wilcoxon rank-sum test are small when the corresponding test statistics $a_{1}$ and *U* are small, Theorem 1 implies that, for univariate numeric data without repeated elements, small Wilcoxon rank-sum test *P*-values force small scXMatch *P*-values (that is, 1D distribution shifts detectable with the Wilcoxon rank-sum test can be detected also with scXMatch). The inverse statement, however, does not hold, as *U* can get large even when $a_{1}$ is small. The reason for this is that the Wilcoxon rank-sum test rejects the null hypothesis $F_{0}=F_{1}$ only if $F_{0}$ is stochastically greater than $F_{1}$ or vice versa, whereas scXMatch can also detect distribution shifts due to differences in spread. A 2D example where scXMatch detects a distribution shift due to a spread difference is shown in Fig. 1h above; Fig. C.5 in Online Appendix C shows a 1D example.

## 5 Discussion

Statistical analysis of high-dimensional data is a common task in single-cell analysis. In this study, we introduced scXMatch—a scalable, biologically validated, and easy-to-use implementation of a statistical test designed to quantify global distribution shifts in high-dimensional data. Instead of relying on dimensionality reduction methods such as PCA or UMAP, scXMatch bases its resulting statistics on a matching calculated in high-dimensional distance space. Unlike commonly used tools for differential feature analysis or clustering-based evaluation, scXMatch only requires a single technical hyperparameter, thereby reducing the risk of confirmation bias. In comparison to approaches like Augur scores, scXMatch assesses global distribution shifts rather than separability. This seemingly subtle difference implies that it can detect distribution shifts even if one cell population lies within a subspace of the other one (thus avoiding false negatives), and that it does not over-emphasize single features which may allow to separate the groups (thus avoiding false positives).

scXMatch implements a runtime- and memory-efficient version of Rosenbaum’s cross matching test (Rosenbaum 2005), making use of an efficient C++-based graph matching algorithm provided by the graph-tool package (Peixoto 2014). To further enhance memory and runtime efficiency, the user can specify a technical hyperparameter *k*, so that the matching is computed on a *k*-NN graph instead of the full distance graph. With the default k=100, scXMatch ran in around 25 minutes on the largest real-world scRNA-seq dataset of our benchmark containing around 70000 cells, with less than 5GB peak RAM usage. On smaller datasets with up to 10000 cells, runtimes were consistently below one minute. Moreover, we showed that the matching construction via the *k*-NN graph implicitly removes outliers and ran ablations which confirmed that scXMatch’s *P*-values remain very stable under different choices of k≥100 as well as subsampling of the minority class.

Aside from these technical aspects, scXMatch’s results cohere with expected biological effects. Experiments on ten scRNA-seq datasets from perturbation studies revealed that, while the permutation-based *E*-distance test is sensitive to distribution shifts, it quickly reaches the smallest possible empirical *P*-value. In contrast, scXMatch yielded *P*-values that induced rankings of the perturbation effects which perfectly aligned with biological expectations in eight of the ten scRNA-seq datasets. Moreover, we observed that scXMatch is faster and more memory-efficient than the *E*-distance test, and that it includes a higher percentage of samples into the final computation of the *P*-values. We also compared scXMatch against other commonly used workflows for identifying distribution shifts in scRNA-seq data and obtained Pareto-optimal results in eight of the ten datasets. Beyond scRNA-seq data, we ran scXMatch on scATAC-seq data, where it returned results that perfectly align with known T cell biology, and on single-cell morphodynamic data of RTMs, where its results highlight the importance of time-lapse *in vivo* imaging to capture the functional state of RTMs.

It is important to stress that the scope of scXMatch is assessing the significance of distribution shifts between experimentally determined cell populations. Beyond this intended usage, scXMatch’s *P*-values are less reliable. For instance, scXMatch should not be used to assess the significance of clusters that are *de novo* inferred from the single-cell data. This would amount to double dipping (Song *et al.* 2024), and scXMatch will thus almost always identify significant distribution shifts. Other dedicated methods exist to assess the significance of clustering results for scRNA-seq data (Grabski *et al.* 2023). Caution also needs to be taken when comparing the results of two highly significant or two non-significant scXMatch tests. When two scXMatch *P*-values are both very small or when they are both non-significant, we observed that the order of the *P*-values does not always reflect the expected biological signal. Moreover, we would like to point out that scXMatch is primarily designed to test *if* a global distribution shift exists, not *why* it exists. Although scXMatch also supports more fine-grained pathway-level analyses (see use case on scATAC-seq data), this is not its primary scope, and we thus recommend users to also rely on complementary approaches to uncover the drivers of the distributions shifts detected with scXMatch.

Our study opens up at least three interesting avenues for future work. Firstly, while scXMatch tests the null hypothesis that two cell populations $X_{0}$ and $X_{1}$ were sampled from the same distribution, a relevant adjacent question is if $X_{1}$ was sampled from a subspace of $X_{0}$. By incorporating outlier detection approaches to assess if all $x\in X_{1}$ are inliers w. r. t. $X_{0}$, scXMatch could be extended to cover also this question. However, such an extension would be *ad hoc* and thus suffer from similar weaknesses as the existing indirect distribution shift quantification approaches which motivated us to develop scXMatch. Hence, an interesting challenge for future work is to design (or rediscover) a non-parametric statistical test which can be used for single-cell data and tests the null hypothesis that $X_{1}$ was sampled from a subspace of $X_{0}$. Secondly, the outlier removal side effect of scXMatch’s *k*-NN graph approach could be exploited more systematically by allowing the user to specify the expected fraction *r* of outliers in the dataset and then setting the parameter *k* such that the relative coverage of the MWMCM computed on the *k*-NN graph is around 1−r. To implement such a strategy, it would be necessary to solve the (to the best of our knowledge) currently unsolved graph-theoretical question how to estimate the size of MWMCMs in *k*-NN graphs. Thirdly, the link between scXMatch and the Wilcoxon rank-sum test for 1D data could be explored in more depth: On the theoretical side, it would be interesting to confirm or refute our conjecture that Theorem 1’s assumption that X does not contain repeated elements can be dropped. On the experimental side, it is worth exploring if scXMatch’s capability to detect distributions shifts in 1D data that are due to differences in spread can be exploited to identify individual genes whose dispersion patterns change between two conditions while their central tendencies remain unchanged.

In sum, we have shown that scXMatch robustly quantifies global distribution shifts in single-cell data of various modalities. Its value to the single-cell community lies in several aspects. Firstly, the tool can be used for almost any kind of single-cell data of all realistic sizes: The test itself is non-parametric, does not expect a specific distribution, and only requires the user to select a distance function to quantify cell dissimilarity. Various distance functions are supported out-of-the-box; custom distances can be provided by the user. Secondly, the concept of matching cells and then evaluating the resulting pairs is intuitively understandable even for users without statistics background. If desired, scXMatch returns the underlying matching, which enables inspection by the user and lends interpretability to scXMatch’s *P*-values. Finally, scXMatch is part of the scverse ecosystem and thus seamlessly interfaces with popular software for single-cell data analysis such as Scanpy (Wolf *et al.* 2018) and ann-data (Virshup *et al.* 2024). It is distributed via the Bioconda package manager (Grüning *et al.* 2018) and can be installed with just one line of code: conda install scxmatch -c conda-forge -c bioconda.

## Supplementary Material

btag706_Supplementary_Data

## Data Availability

We obtained scRNA-seq data from pertpy’s data module (https://pertpy.readthedocs.io/en/stable/api/datasets_index.html), scATAC-seq data from https://doi.org/10.5281/zenodo.7058382 (file MimitouSmibert2021.zip), and macrophage morphodynamics data from https://doi.org/10.5281/zenodo.13929787 and https://github.com/MiriamSchnitzerlein/MacrophageMorphodynamics.
