# Supplementary material for: Quantifying distribution shifts in single-cell data with scXMatch
Source: Bioinformatics. 2026 Oct 3;42(10):btag706. doi: 10.1093/bioinformatics/btag706 (PMC13645430; doi:10.1093/bioinformatics/btag706)
Supplement: Supplementary file 1 [file btag706_supplementary_data.pdf]

Quantifying distribution shifts in single-cell data with scXMatch
Appendices

Anna Möller[1,2], Miriam Schnitzerlein[5,6,7], Eric Greto[2,3,4], Vasily Zaburdaev[5,6], Stefan Uderhardt[2,3,4], and David B. Blumenthal[1]

[1] Biomedical Network Science Lab, Department of Artificial Intelligence in Biomedical Engineering, Friedrich-Alexander-Universität Erlangen-Nürnberg (FAU), Erlangen, Germany
[2] Department of Medicine 3 – Rheumatology and Immunology, Friedrich-Alexander-Universität Erlangen-Nürnberg (FAU) and Universitätsklinikum Erlangen, Erlangen, Germany
[3] Deutsches Zentrum für Immuntherapie, FAU, Erlangen, Germany
[4] Exploratory Research Unit, Optical Imaging Competence Center Erlangen, FAU, Erlangen, Germany
[5] Department of Biology, FAU, Erlangen, Germany
[6] Max-Planck-Zentrum für Physik und Medizin, Erlangen, Germany
[7] Max Planck Institute for the Science of Light, Erlangen, Germany

A Detailed methods

A.1 Rosenbaum's cross-matching test

Our tool scXMatch is based on Rosenbaum's cross-matching test [15]. To make this article self-contained, we here provide a high-level explanation of this test. Further details and proofs can be found in the original article.

The cross-matching test is designed for the following setup: We are given $N \geq 4$ samples $i \in \{1, \ldots, N\}$ with binary condition labels $y_i \in \{0, 1\}$. Of the N samples, $n \geq 2$ belong to a target condition $y_i = 1$ (i. e., $\sum_{i=1}^{N} y_i = n$) and the remaining $m = N - n \geq 2$ samples belong to a control condition $y_i = 0$. All samples are associated with multivariate data $\mathbf{x}_i$ from some data space $\mathbb{X}$ for which a suitable distance measure $d : \mathbb{X} \times \mathbb{X} \to \mathbb{R}_{\geq 0}$ is available. In the context of this article, we focus on the case where the samples are cells, $\mathbf{x}_i$ contains multivariate numeric data (e.g., gene expression or chromatin accessibility from scRNA-seq or scATAC-seq studies), and the condition labels y_i encode experimental conditions (e.g., wild type versus knockout, diseased versus healthy, treated versus untreated, etc.). However, in principle, the cross-matching test can be applied to any data as long as a distance measure d and binary condition labels are available.

Let $[N] = \{1, \ldots N\}$, $\mathcal{X} = \{\mathbf{x}_i \mid i \in [N]\}$, $\mathcal{X}_0 = \{\mathbf{x}_i \in \mathcal{X} \mid y_i = 0\}$, and $\mathcal{X}_1 = \{\mathbf{x}_i \in \mathcal{X} \mid y_i = 1\}$. Moreover, let F_0 and F_1 be two distributions over $\mathbb{X}$ such that $\mathcal{X}_0$ is sampled from F_0 and $\mathcal{X}_1$ is sampled from F_1. The null hypothesis of the cross-matching test is that $F_0 = F_1$. The test makes no assumptions on the distribution $F_0 = F_1$ under the null hypothesis, i. e., the distribution of the data under the null hypothesis may be unknown.

To compute its test statistic, the cross-matching test first constructs a weighted complete undirected graph $G = ([N], E, w)$ over the samples in the dataset, with the weight $w_{ij} \geq 0$ of an edge $ij \in E = \binom{[N]}{2}$ defined as the distance $w_{ij} = d(\mathbf{x}_i, \mathbf{x}_j)$ between the involved samples' data records. For even N (the case where N is odd is discussed in the next subsection), the cross-matching test then computes a minimum-weight perfect matching (MWPM) M for G, i. e., an edge set $M \subset E$ such that all nodes $i \in [N]$ are covered by exactly one edge $ij \in M$ (perfect matching) and M minimizes the overall edge weight $w(M) = \sum_{ij \in M} w_{ij}$ among all perfect matchings. For a fixed MWPM M and $l \in \{0, 1, 2\}$, let $A_l = |\{ij \in M \mid y_i + y_j = l\}|$ be the number of edges in M such that l incident nodes belong to condition 1. Note that $A_0 + A_1 + A_2 = |M|$ holds by construction and that we moreover have $N = 2|M|$, $n = A_1 + 2A_2$, $A_2 = (n - A_1)/2$, and $A_0 = |M| - (n + A_1)/2$, since M is a perfect matching (i.e, A_1 determines A_0 and A_2). The cross-matching test uses A_1 as its test statistic — the number of crossing edges $ij \in M$ which connect samples i and j with different condition labels $y_i \neq y_j$. Under the null hypothesis $F_0 = F_1$, A_1 is distributed as follows:

$$Pr(A_1 = a_1) = \binom{N}{n}^{-1} \frac{|M|!}{a_0! a_1! a_2!} 2^{a_1} \tag{A.1}$$

To see why this is the case, we count the number of condition label assignments $(y_i)_{i=1}^N$ such that $\sum_{i=1}^N y_i = n$. There are $\binom{N}{n}$ such assignments, and, under the null hypothesis, they are independent of the matching M and thus all have an equal probability $\binom{N}{n}^{-1}$. Now we ask: How many condition label assignments are there such that $A_1 = a_1$ holds in addition to $\sum_{i=1}^N y_i = n$? To answer this question, consider one of the $|M|!$ different orderings

$$O = (\underbrace{i_1 j_1, \ldots, i_{a_0} j_{a_0}}_{O_0}, \underbrace{i_{a_0+1} j_{a_0+1}, \ldots, i_{a_0+a_1} j_{a_0+a_1}}_{O_1}, \underbrace{i_{a_0+a_1+1} j_{a_0+a_1+1}, \ldots, i_{|M|} j_{|M|}}_{O_2}) \tag{A.2}$$

of the edges $ij \in M$. Given O, we can construct a condition label assignment with $\sum_{i=1}^N y_i = n$ and $A_1 = a_1$ by setting $y_i = 0$ for all samples i covered by O_0, $y_i = 1$ for all samples i covered by O_2, and flipping a coin for each edge $ij \in O_1$ to decide if we set $y_i = 0$ and $y_j = 1$ or $y_i = 1$ and $y_j = 0$. There are 2^{a_1} different outcomes of the coin flipping subroutine, and, given a fixed outcome of this subroutine, two orderings which are identical up to permutation of O_0, O_1, and O_2 yield the same condition label assignment. This implies that, under the null hypothesis $F_0 = F_1$, A_1 is distributed according to Equation (A.1).

If $F_0 \neq F_1$, data elements $\mathbf{x}_i$ and $\mathbf{x}_j$ are sampled from different distributions for samples i and j with $y_i \neq y_j$, and so the weights w_{ij} are, on average, larger for crossing edges $ij \in E$ with $y_i \neq y_j$ than for pairs of samples with identical condition labels. Since the MWPM M prioritizes edges with small weights, the number A_1 of crossing edges in M is hence expected to be smaller under the alternative hypothesis $F_0 \neq F_1$ than under the null hypothesis. Consequently, we can reject the null hypothesis if the following P-value is small:

$$Pr(A_1 \leq a_1) = \sum_{a_1'=0}^{a_1} \binom{N}{n}^{-1} \frac{|M|!}{a_0'! a_1'! a_2'!} 2^{a_1'} \tag{A.3}$$

Rosenbaum [15] further shows that, under the null hypothesis, A_1 has an expected value and a variance of

$$\mathbb{E}(A_1) = \frac{nm}{N-1} \qquad var(A_1) = \frac{2n(n-1)m(m-1)}{(N-3)(N-1)^2}, \tag{A.4}$$

which, for a given a_1, allows us to compute a z-score

$$z(a_1) = (a_1 - \mathbb{E}(A_1))/\sqrt{var(A_1)} \tag{A.5}$$

that is negative when the null hypothesis is rejected. Moreover, we introduce an effect-strength ratio

$$R = 1 - \frac{\min\{a_1, \mathbb{E}(A_1)\}}{\mathbb{E}(A_1)} \in [0, 1], \tag{A.6}$$

which equals 0 when the observed number of cross matches a_1 is larger or equal to the expected value under the null hypothesis and is close to 1 when the null hypothesis is rejected. Moreover, R is independent of the sample size N, since both a_1 and $\mathbb{E}(A_1)$ scale linearly with N. R is hence similar to an effect size measure in the sense that it constitutes a sample size-invariant measure of the magnitude of the distribution shift.

A.2 Rosenbaum's handling of odd sample numbers

If N is odd, the graph $G = ([N], E, w)$ as introduced above does not have a perfect matching and so the probability distributions and P-values in Equations (A.1) and (A.3) are not well-defined. To handle this case, Rosenbaum [15] suggests the following procedure:

1. Add dummy node ϵ to G and connect it to all real nodes $i \in [N]$ via edges of weight $w_{i\epsilon} = 0$.
2. Compute a MWPM M_ϵ in the resulting augmented graph G_ϵ.
3. Discard the unique sample i such that $i\epsilon \in M_\epsilon$ and run the cross-matching test on graph G' containing the remaining $N-1$ samples (note that this also implies decreasing n by 1 if $y_i = 1$ holds for the discarded sample i).

We now provide an equivalent variant of this procedure, which will allow us to derive the speedup presented in the next subsection. Our variant is based on the notion of minimum-weight maximum-cardinality matchings (MWMCM). An MWMCM of a weighted undirected graph $G = ([N], E, w)$ is an edge set $M \subset E$ which covers each node $i \in [N]$ at most once, has maximum cardinality among all such edge sets (maximum-cardinality matching), and minimizes $w(M)$ among all maximum-cardinality matchings. MWMCMs exist for all weighted undirected graphs $G = ([N], E, w)$ and are perfect matchings whenever $G = ([N], E, w)$ has a perfect matching. We now make the following observation:

Observation 1 *Let N be odd and G, G', G_ϵ, and M_ϵ be constructed as detailed above and let M' be a MWPM for G'. Then M' is a MWMCM for G.*

Proof. By construction and since N is odd, any perfect matching for G' is a maximum-cardinality matching for G, and hence so is M'. Assume that there is a maximum-cardinality matching $M^\star$ for G with $w(M^\star) < w(M')$. Let $i^\star \in [N]$ be the unique sample left uncovered by $M^\star$ and let $M^\star_\epsilon = M^\star \cup \{i^\star \epsilon\}$. Then $M^\star$ is a perfect matching for $G^\star$ with $w(M^\star_\epsilon) = w(M^\star) + w_{i^\star \epsilon} = w(M^\star) < w(M') = w(M_\epsilon)$, contradicting the choice of M_ϵ. □

Observation 1 leads to the following equivalent variant of Rosenbaum's procedure to handle odd N:

1. Compute a MWMCM M for G and collect the set $C(M) = \{i \in [N] \mid \exists e \in M : i \in e\}$ of samples covered by an edge $e \in M$.
2. Update the constants N, n, and m as follows: $N = |C(M)|$, $n = \sum_{i \in C(M)} y_i$, and $m = N - n$.
3. Compute the P-value and the z-score according to Equations (A.3) to (A.5), using the updated values of N, n, and m.

A.3 A more resource-efficient variant of the test based on k-nearest neighbor graphs

A major bottleneck of the original version of the cross-matching test is that it computes the matching M in the complete graph over the N samples. This implies that $O(N^2)$ calls to the distance function d are required to construct G and that we need $O(N^2)$ space to keep G in memory. While this lack of efficiency may be acceptable when N is small (e.g., because the samples correspond to patients as in the original paper's motivating example), it makes it impossible to use the cross-matching test out-of-the-box for the comparison of cell populations in single-cell datasets with thousands of cells.

To overcome this shortcoming, we observe that the alternative procedure to handle odd N explained at the end of the previous subsection can be run on arbitrary graphs G and not only when G is a complete weighted graph with an odd number of nodes. For our speedup of the cross-matching test, we hence simply construct G as a k-NN graph on $[N]$, where neighborhoods are defined in terms of the distance measure d. Although exact k-NN graph construction also requires $O(N^2)$ distance computations, approximate k-NN graphs can be constructed efficiently, e.g., via the widely used NN-descent method [2]. Since, under the null hypothesis, connectivity in the k-NN graph is independent of the condition labels y_i, Equation (A.1) remains valid.

The main difference with respect to the original version is that there may now be several samples that are discarded before computing the P-value. Let

$$N_{\text{drop}} = N - |C(M)| \geq 0 \quad \text{and} \quad cov = \frac{|C(M)|}{2\lfloor N/2 \rfloor} \in [0, 1] \tag{A.7}$$

denote, respectively, the number of discarded samples and the relative sample coverage of the matching M used towards the P-value computation. Smaller k lead to a larger N_{drop}, a smaller cov, and a more substantial gain in runtime and memory efficiency. As in the original version's procedure for handling odd N, under the null hypothesis, samples are discarded or retained independently of the condition labels y_i, and the discarded samples can be viewed as outliers in the multivariate dataset $\mathcal{X}$ with respect to the distance measure d. We have $cov = 1$ whenever M is a perfect matching (for even N) or a matching covering all but one of the N samples (for odd N).

A.4 Implementation details

Since scXMatch is implemented as a Python package coherent to the guidelines of the scverse project [19], the input data is expected to be provided in anndata format [20]. Algorithmically, scXMatch is structured into three parts: first, the construction of the distance-weighted graph $G = ([N], E, w)$ with edge weights $w_{ij} = d(\mathbf{x}_i, \mathbf{x}_j)$, second, the computation of a MWMCM M in G, and third, the computation of the statistics given the resulting matching M and the group assignments $\mathbf{y}$. We rely on the following software packages for each of the steps:

Construction of distance-weighted graph. The package offers two different approaches for this purpose. The user can decide to either construct a complete graph, or a k-NN graph. The creation of the complete graph can be performed either on a CPU or a GPU. On a CPU, the package relies on `scipy.spatial.distance.cdist` [21]. If a GPU and a functioning `CuPy` [11] installation are available, the computation is automatically run on the GPU. Calculating the distance matrix on a GPU leads to a significant speedup and is therefore strongly recommended for constructing the complete graph. The `cupyx.scipy.spatial.distance.cdist` implementation holds slightly fewer options for the chosen metric, but `sqeuclidean`, which scXMatch uses as a default for scRNA-seq data, is implemented. If the user decides to construct a k-NN graph, `scanpy.pp.neighbors` [23] is used. To ensure that the dissimilarity is calculated on the actual omics data and not on a lower-dimensional representation, we set the parameter `n_pcs=0`. `scanpy`'s k-NN graph construction relies on the NN-descent algorithm [2]. The resulting (sparse) distance-weighted adjacency matrix $(w_{ij})_{ij\in E}$ of G is efficiently stored as a `scipy.sparse.csr_matrix`.

Computation of MWMCM in distance-weighted graph. To compute an MWMCM M in G, we use `graph-tool` [13]. This Python package is especially suited for large-scale graph operations, as it is mostly implemented in C++. When constructing an undirected, weighted graph from a `scipy.sparse.csr_matrix` holding the weights, the `graph_tool.Graph` constructor ignores the lower diagonal of the matrix, assuming that the adjacency matrix is symmetric. As strictly speaking, the matrix constructed from the k-NN graph distances is not undirected — a node x_i can be in a node x_j's k nearest neighbors while x_j not being in x_i's, which would lead to $w_{ij} = \infty$ but $w_{ji} < \infty$ in the weighted adjacency matrix of the k-NN graph — we thus modified the weights as

$$w'_{ij} = \min\{w_{ij}, w_{ji}\} \tag{A.8}$$

to construct an undirected graph that accurately preserves the neighboring relationships. The MWMCM M is retrieved with `max_cardinality_matching`. This function internally calls the C++ Boost Graph Library implementation of [3]'s algorithm to compute maximum-weight matchings, which runs in $O(N^3)$ time. To use this function, we hence have to transform our MWMCM instance into an equivalent instance of the maximum weight matching problem. We achieve this by further transforming the edge weights as follows:

$$w''_{ij} = \max_{i'j'\in E} w'_{i'j'} + 1 - w'_{ij} \tag{A.9}$$

Computation of P-values and z-scores. Given the matching M and the condition labels y_i, we implemented the remaining computations with basic Python and `numpy` functionalities. Since the computation of the final P-values leads to large numerators (see Equation (A.3)), we calculate the summands in logarithmic space and then aggregate the exponentials.

A.5 Datasets used for benchmarking

Simulated data. For the experiments to determine runtime and memory requirements, we simulate data and matching construction for all possible combinations of $N \in \{500, 1000, 2000, 5000, 10000, 20000, 50000\}$, $h = \{10, 500, 1000, 2000\}$, and $k \in \{10, 20, 50, 100, 200, 500, 1000, 2000, 5000\}$. The values for N and h were chosen such that they mimic common sizes of scRNA-seq datasets after pre-processing (e.g., filtering for highly variable genes). The chosen values for k aim at investigating how the matching coverage behaves for both small but efficient and bigger but less efficient k.

– Random Gaussian data: Given the different parameter combinations, the data was randomly drawn from a normal distribution using NumPy's `random.normal` function.

- Splatter data: To mimic the characteristics of scRNA-seq data, we used the R package Splatter [24] with default parameters and `dropout.type="experiment"`. Splatter models biological variation and differential expression, and most importantly applies an expression-dependent dropout to simulate sparsity. All resulting datasets contain at least 90% zero counts.

Perturbation scRNA-seq data. We downloaded all datasets via the Python package `pertpy`. We `log1p`-transformed the data and selected the 2000 most variable genes using `scanpy`. The datasets have the following characteristics:

- The Bhattacherjee data contains scRNA-seq data from different mice [1]. Three cohorts were on a steady level of cocaine for 15 days. Then, two cohorts experienced withdrawal for 48 hours and 15 days, respectively. From each of the groups, cells from the prefrontal cortex were collected and sequenced. We subset the dataset to the cell types with over 200 cells in each group, namely astrocytes, endothelial cells, and excitatory neurons. The number of cells per type and group is shown in Table A.1. As we use the maintenance status as control, the perturbation strength is expected to be higher for the 15-day cohort.

Table A.1: Number of cells per type and group in the Bhattacherjee subsets.

Name	Cell type	Control	48h	15d
Bhattacherjee 1	Astrocytes	216	270	213
Bhattacherjee 2	Endothelial cells	478	477	753
Bhattacherjee 3	Excitatory neurons	1264	1688	4789

- The McFarland dataset was generated using a novel MIX-Seq approach [7]. Well-characterized cancer cell lines were treated with 13 different drugs (including targeted therapies, general cytotoxics, and highly selective compounds) and sequenced after 6 and 24 hours. We filter for (cell line, treatment) combinations with more than 200 cells for each time point and are left with 5 subsets, which include thyroid anaplastic carcinoma cells (CAL62), tongue squamous cell carcinoma cells (BICR31), and ovarian cancer cells (OAW42 and IGROV1), which were treated with either the strongly selective tool compound BRD3379, or the targeted cancer therapies Idasanutlin or Trametinib. We name them as shown in Table A.2. Perturbation strength corresponds to increasing drug exposure time.

Table A.2: Number of cells per type and group in the McFarland subsets.

Name	Cell line	Treatment	control	6h	24h
McFarland 1	BICR31	Idasanutlin	683	252	277
McFarland 2	BICR31	Trametinib	683	217	337
McFarland 3	CAL62	BRD3379	411	223	322
McFarland 4	IGROV1	BRD3379	457	206	274
McFarland 5	OAW42	BRD3379	340	201	296

- The Norman dataset originates from a pooled CRISPR-based screen designed to investigate the effects of single versus pairwise gene perturbations in K562 cells [10]. Upon grouping the dataset into control, single-guide, and dual-guide conditions, we obtained 11,835, 57,735, and 41,685 cells, respectively. For the benchmark described in Section 3.2 of the main article, we subset the dataset and compared single-guide overexpression of UBASH3B (4797 cells) and dual-guide overexpression of UBASH3B and CNN1 (401 cells) to control (11,835 cells). We selected this subset because it has the highest cell count of the combinations that are reported as strongly synergetic in the original publication [10], so that we can safely assume that the single-guide overexpression is the weaker perturbation than the dual-guide overexpression. For the outlier analysis in described in Section 3.3 of the main article and the runtime analysis

whose results are shown in Figure C.3, we used the entire dataset, as these analyses are independent of the group labels.
- The Schiebinger dataset was collected over an 18-day time course to study transcription factor–mediated cellular reprogramming [16]. Between 1,956 and 11,088 mouse embryonic fibroblasts were profiled every 12 hours using single-cell RNA sequencing, resulting in a total of 163,532 cells across 35 time points. Perturbation strength follows the day of measurement after cellular reprogramming.

Perturbation scATAC-seq data. We downloaded the Mimitou scATAC-seq data [8] from scPerturb [12] and used the provided gene scores. The data consists of primary human CD4+ T cells subjected to targeted CRISPR–Cas9 perturbations of key genes involved in T cell receptor (TCR) signaling, namely CD3E (621 cells), CD4 (1167 cells), ZAP70 (1253 cells), and NFKB2 (1024 cells), as well as a double knockout of CD3E and CD4 (1167 cells) and a control set (1112 cells). Like for the scRNA-seq data, we `log1p`-transform the gene scores and subset the features to the 2000 most variable genes. For the pathway-level analysis, we subset the features to 195 genes retrieved from `gseapy` (version 1.2.1), library `MSigDB_Hallmark_2020`, and key `TNF-alpha Signaling via NF-kB`. Before running the E-distance test, we subsampled all groups to the size of the majority class, leading to a total of 3726 cells, and performed PCA on the dataset (as advised, see below).

Macrophage morphodynamics. The *in vivo* morphodynamics data was retrieved from two-dimensional microscopy videos of resident tissue macrophages under different stimuli. To derive the features, the macrophages were segmented and tracked over time. Details on the imaging and feature extraction can be found in the original publication [17].

A.6 Baseline methods

The E-distance test. The E-distance test is a permutation-based approach to detect distribution shifts in multivariate data which has been suggested as a measure to quantify perturbation effects by Peidli *et al.* [12]. When comparing n data profiles $\mathbf{x} \in \mathcal{X}_1$ from a target condition to m data profiles $\mathbf{x} \in \mathcal{X}_0$ from control condition, the test first subsamples the majority class to ensure $|\mathcal{X}_1| = n = m = |\mathcal{X}_0|$, then linearly transforms the data using principal component analysis (PCA), and subsequently computes the E-distance

$$E(\mathcal{X}_0, \mathcal{X}_1) = \frac{2}{nm} \sum_{\mathbf{x}_i \in \mathcal{X}_0} \sum_{\mathbf{x}_j \in \mathcal{X}_1} \|\widehat{\mathbf{x}}_i - \widehat{\mathbf{x}}_j\|_2 - \frac{1}{n^2} \sum_{\mathbf{x}_i \in \mathcal{X}_0} \sum_{\mathbf{x}_j \in \mathcal{X}_0} \|\widehat{\mathbf{x}}_i - \widehat{\mathbf{x}}_j\|_2 - \frac{1}{m^2} \sum_{\mathbf{x}_i \in \mathcal{X}_1} \sum_{\mathbf{x}_j \in \mathcal{X}_1} \|\widehat{\mathbf{x}}_i - \widehat{\mathbf{x}}_j\|_2, \quad \text{(A.10)}$$

where $\widehat{\mathbf{x}}$ is the D-dimensional PCA representation (suggested default: $D = 50$ [12]) of data profile $\mathbf{x}$. The same computation is carried out K times with shuffled class labels (suggested default: $K = 10000$ [12]), yielding an empirical background distribution $\{E(\mathcal{X}_0^{(k)}, \mathcal{X}_1^{(k)})\}_{k=1}^{K}$ of E-distances that leads to the empirical P-value

$$P = \frac{1 + |\{k \in \{1, \dots, K\} \mid E(\mathcal{X}_0, \mathcal{X}_1) \leq E(\mathcal{X}_0^{(k)}, \mathcal{X}_1^{(k)})\}|}{K + 1} \quad \text{(A.11)}$$

which is small whenever the E-distances for the real condition labels are larger than expected by chance. We ran the E-distance test with $K \in \{10000, 20000\}$ and both with and without subsampling of the majority class to ensure $n = m$, and included both the empirical P-values and the raw E-distances $E(\mathcal{X}_0, \mathcal{X}_1)$ into our tests.

Number of differentially expressed genes. We identify DEGs between each test group and the reference group with these four approaches:

- The Wilcoxon test is a non-parametric statistical test that compares the ranks of gene expression values between groups without assuming normal distribution [22]. It is commonly used for scRNA-seq data due to its robustness to outliers and ability to handle non-Gaussian distributions.
- Memento interprets scRNA-seq as a hypergeometric sampling process, explicitly decoupling biological variability from measurement [5]. It derives closed-form estimators for the mean, residual variance, and gene-gene correlation of expression to compute P-values for differential mean, differential variability, and differential correlation. We ran Memento using its `binary_test_1d` wrapper with a capture

rate of $q = 0.07$, consistent with the authors' recommended default for 10X Chromium data. Whenever replicate annotations were available (Schiebinger and Bhattacherjee data), we also generated results using the `ht_1d_moments` wrapper with the replicate information as covariate. The performance of `binary_test_1d` was better regarding all benchmarking metrics, hence we discarded the replicate-aware results.

- The edgeR method is based on negative binomial models and was originally developed for bulk RNA-seq [14]. It accounts for biological variability and is well-suited for datasets with small sample sizes or overdispersed count data. Since edgeR is designed for bulk data, we randomly generated pseudo-bulk samples of 100 cells before running it, constrained to contain only cells from one condition and one biological replicate whenever replicate annotations were available (this was the case only for the Schiebinger and Bhattacherjee data).
- PyDESeq2 is a Python implementation of the DESeq2 algorithm, which models gene counts using a negative binomial distribution and applies shrinkage estimators for dispersion and fold changes [6,9]. It is designed to handle count-based expression data and provides robust statistical testing even in the presence of noise. Since PyDESeq2 is also designed for bulk data, we used the same pseudo-bulking strategy as for edgeR.

Augur scores. Augur is a cell type prioritization method that quantifies the separability of perturbed versus control cells within one cell type using supervised classification [18]. It trains a classifier (by default a random forest) to distinguish between conditions and evaluates performance via cross-validated AUC scores. High Augur scores indicate that transcriptional profiles under a perturbation are readily distinguishable from controls, making it a useful metric for comparing the magnitude of perturbation effects.

We used `pertpy`'s [4] implementation of the E-distance, the Wilcoxon test, edgeR, PyDESeq2, and the Augur scores. Memento was run using the code from the original publication [5].

A.7 Evaluation metrics

Runtime and memory. We recorded runtime and memory requirements for all 56 simulated datasets with $(N, h) \in \{500, 1000, 2000, 5000, 10000, 20000, 50000\} \times \{10, 500, 1000, 2000\}$ and for each of the 9 different examined values $k \in \{10, 20, 50, 100, 200, 500, 1000, 2000, 5000\}$. All jobs were run on shared cluster nodes equipped with 2 × AMD EPYC 7502 CPUs (64 cores total). Each job was allowed to use 16 CPU cores and up to 512 GB of RAM. We recorded the runtimes using Python's `time` module. Peak memory consumption was measured as the maximum resident set size (RSS) reported by the Linux `resource` module (`ru_maxrss`) over the full process, including all child processes spawned during execution, and reported in gigabytes.

Monotonicity w. r. t. perturbation strength. Let $(\mathcal{X}_0, \mathcal{X}_1, \ldots, \mathcal{X}_L)$ be one of the scRNA-seq perturbation datasets described above, where the set $\mathcal{X}_0$ contains the cells from the control condition, the sets $(\mathcal{X}_l)_{l=1}^L$ contain corresponding cells from the L perturbed conditions, and l increases with ground-truth perturbation strength. Moreover, let s be one of the tested distribution shift scores (i. e., scXMatch P-value, Augur score, E-distance, or number of differentially expressed genes) and $(s_{0,l})_{l=1}^L$ be the values obtained with s when comparing cells in $\mathcal{X}_0$ to cells in $\mathcal{X}_l$, for $l \in [L]$. We define a monotonicity index

$$\mathrm{MI}(s) = \binom{L}{2}^{-1} \sum_{l=1}^{L-1} \sum_{l'=l+1}^{L} \texttt{isStrongerOrEqual}(s_{0,l'}, s_{0,l}) \in [0, 1], \tag{A.12}$$

where $\texttt{isStrongerOrEqual}(s_{0,l'}, s_{0,l}) \in \{0, 1\}$ is set to 1 if and only if $s_{0,l'}$ is indicative of a stronger or equally strong distribution shift than $s_{0,l}$ (i. e., `isStrongerOrEqual` = `isLessOrEqual` for the P-values computed by scXMatch and `isStrongerOrEqual` = `isGreaterOrEqual` for the number of differentially expressed genes and the Augur scores). $\mathrm{MI}(s)$ counts the perturbation strength pairs where the differences in global distribution shifts w. r. t. the control condition quantified by s matches the biological expectation and divides this count by the total number of perturbation strength pairs. Values close to 1 indicate that the distribution shift score s is consistent with the biological expectation. We decided to assess monotonicity rather than strict monotonicity in order not to punish scores in situations where the effect of the biological perturbation is either very weak or very strong across all perturbation strengths l. To prevent that $\mathrm{MI}(\cdot)$ overemphasizes tiny differences, $\texttt{isStrongerOrEqual}(\cdot, \cdot)$ rounds the compared scores to two significant figures before comparing them (e. g., 0.0123 and 0.0121 are treated as equal).

False positive rate estimation for scXMatch and signal-stronger-than-noise ratio computation. As before, let $(\mathcal{X}_0, \mathcal{X}_1, \dots, \mathcal{X}_L)$ be one of the scRNA-seq perturbation datasets. For $l = 0, \dots, L$, we randomly sampled subsets $\mathcal{X}_l^q$ from $\mathcal{X}_l$ by assigning cells to $\mathcal{X}_l^q$ with probability $q \in Q = \{0.1, 0.3, 0.5\}$. We then used all benchmarked distribution shift scores s to compare cells in $\mathcal{X}_l^q$ and $\mathcal{X}_l \setminus \mathcal{X}_l^q$, obtaining distribution shift scores $s_{l_q, \overline{l_q}}$. When s is the P-value computed by scXMatch, the scores $s_{l_q, \overline{l_q}}$ can be used to assess the false positive rate by counting the scores that reach statistical significance. We carried out this analysis using 0.05 as significance cutoff for both raw and adjusted P-values that were corrected for multiple testing within each test group via the Benjamini-Hochberg procedure, with the number of tests set to $(L+1) \cdot |Q|$. Additionally, we assessed for all benchmarked distribution shift scores s if they are systematically stronger for comparisons between different conditions than for comparisons within the same condition. To this end, we computed the signal-stronger-than-noise ratio

$$\mathrm{SSNR}(s) = (L \cdot |Q|)^{-1} \sum_{l=1}^{L} \sum_{q \in Q} \texttt{isStrongerOrEqual}(s_{0,l}, s_{l_q, \overline{l_q}}) \in [0, 1], \tag{A.13}$$

which assumes values close to 1 if the score s is consistently stronger or equal when comparing cells from a perturbed condition to cells from the control condition than when comparing cells from two subsets of the same perturbed condition.

Robustness. Finally, we assessed how robust the different distribution shift scores are when evaluated on different subsets of the control and perturbed conditions. For $l = 0, \dots, L$, we randomly sampled subsets $\mathcal{X}_l^q$ from $\mathcal{X}_l$ by assigning cells to $\mathcal{X}_l^q$ with probability $q \in Q = \{0.1, 0.3, 0.5\}$. For all pairs of subsampling rates $(q, q') \in Q \times Q$ and all perturbed conditions $l = 1, \dots, L$, we then used the benchmarked distribution shift scores s to compare cells in $\mathcal{X}_0^q$ and $\mathcal{X}_l^{q'}$, obtaining distribution shift scores $s_{0_q, l_{q'}}$. Let $s_{0,l}$ be the corresponding distribution shift score obtained when comparing $\mathcal{X}_0$ and $\mathcal{X}_l$ without subsampling. Then, we computed the subsampling robustness as the average complement of the relative error

$$\mathrm{SR}(s) = (L \cdot |Q|^2)^{-1} \sum_{l=1}^{L} \sum_{q \in Q} \sum_{q' \in Q} \left(1 - \frac{|s_{0_q, l_{q'}} - s_{0,l}|}{s_{0,l} + \epsilon}\right) \leq 1, \tag{A.14}$$

where ϵ is a small constant to avoid division by zero if $s_{0,l} = 0$. As SR is only comparable for different scores s if the directions of the scores are all the same and scXMatch's P-value is the only score included in the benchmark for which smaller means stronger, we transformed the scXMatch P-values as $s = 1 - P$ before computing SR. All other scores were used as they are.

Pareto optimality. Equipped with the performance metrics $\mathrm{MI}(\cdot)$, $\mathrm{SSNR}(\cdot)$, and $\mathrm{SR}(\cdot)$, we determined Pareto optimality for all pairs of distribution shift scores s and scRNA-seq datasets $\mathcal{X}$ by assessing if s is Pareto-dominated by another distribution shift score s' on $\mathcal{X}$. The score s' Pareto-dominates s on $\mathcal{X}$ if and only if s' is better than s with respect to at least one of the three performance metrics (better = larger for all three metrics) and not worse with respect to any of them. To avoid overemphasizing tiny differences, we round the performance metrics to two significant figures before comparing them.

Local outlier factors. The LOF is a widely used density-based outlier score with LOF ≈ 1 for inliers and LOF > 1 for outliers. To compute LOFs for the samples from the Norman dataset, we used scikit-learn's `LocalOutlierFactor`. We applied the same value of k and distance metric as used in the corresponding run of scXMatch. Since the module inherently returns negative LOFs, we inverted them to improve the interpretability of the results.

B Proof of Theorem 1

Throughout this section, we assume that the assumptions of Theorem 1 hold. We sort the $N = n + m$ elements in $\mathcal{X} = \mathcal{X}_0 \cup \mathcal{X}_1 \subset \mathbb{R}$ in increasing order, obtaining an ordering $\{x_1, \dots, x_N\}$. We structure the proof of Theorem 1 into Lemmata B.1 to B.3.

Lemma B.1. *Let G be either the complete distance-weighted graph over $[N]$ or a k-NN graph with $k \geq 2$, and let M be a MWMCM in G. For each edge $ij \in M$, we define the unique orientation (i, j) such that $i < j$ in our ordering $\{x_1, \ldots, x_i, \ldots, x_j, \ldots, x_N\}$ of $\mathcal{X}$. It holds that $j = i + 1$ for all $ij \in M$.*

Proof. Since all k-NN graphs with $k \geq 2$ contain all consecutive edges of the form $i(i + 1)$, it suffices to show the lemma for the case where G is the complete distance-weighted graph over $[N]$. Aiming for a contradiction, we assume that the MWMCM M contains an edge $ij \in M$ with orientation (i, j) such that $I(i, j) = \{l \in \mathbb{N} \mid i < l < j\} \neq \emptyset$. Of all such non-consecutive edges, we pick an edge $i^\star j^\star \in M$ with orientation $(i^\star, j^\star)$ that minimizes $|I(i^\star, j^\star)|$. Let $I^\star = I(i^\star, j^\star)$. We distinguish three cases:

1. There is an index $l \in I^\star$ such that l is not covered by any edge in M.
2. There is an index $l \in I^\star$ such that there is an edge $ls \in M$ for some $s < i^\star$ or $s > j^\star$.
3. $|I^\star|$ is even and all consecutive edges $(i^\star + 1)(i^\star + 2), \ldots, (i^\star + |I^\star| - 1)(i^\star + |I^\star|)$ are contained in M.

Since $(i^\star, j^\star)$ minimizes $|I(\cdot, \cdot)|$ over all non-consecutive edges in M, these are all possible cases: there is no pair of non-consecutive indices $l, l' \in I$ such that $ll' \in M$. In all three cases, we can construct a maximum-cardinality matching M' that has a lower weight then M, contradicting the choice of M as a MWMCM: In case 1, we set $M' = M \setminus \{i^\star j^\star\} \cup \{i^\star l\}$. In case 2, we define $M' = M \setminus \{i^\star j^\star, ls\} \cup \{si^\star, lj^\star\}$ if $s < i^\star$ and $M' = M \setminus \{i^\star j^\star, ls\} \cup \{i^\star l, j^\star s\}$ if $s > j^\star$. In case 3, we construct M' as $M' = M \setminus (\{i^\star j^\star\} \cup \{(i^\star + 1)(i^\star + 2), \ldots, (i^\star + |I^\star| - 1)(i^\star + |I^\star|)\}) \cup \{i^\star(i^\star + 1), (i^\star + 2)(i^\star + 3), \ldots, (i^\star + |I^\star| - 2)(i^\star + |I^\star| - 1), (i^\star + |I^\star|)j^\star\}$. □

Lemma B.2. *Let $\{R_1, \ldots, R_l, \ldots, R_r\}$ be the unique partition of the ordering $\{x_1, \ldots, x_N\}$ into inclusion-maximal runs of elements with the same condition labels (e. g., $R_1 = \{x_1, \ldots, x_{b_1}\}$, where b_1 is the unique index such that $y_i = y_1$ holds for all indices $i \in \{1, \ldots, b_1\}$ but not for the index $i = b_1 + 1$). Then it holds that $a_1 \leq r - 1$.*

Proof. From Lemma B.1, we know that all edges in M correspond to consecutive pairs of the form $(i, i+1)$. At the same time, the only consecutive pairs $(i, i + 1)$ with $y_i \neq y_{i+1}$ occur at the $r - 1$ boundaries between the r runs R_l. Thus, at most $r - 1$ edges in M are crossing edges, which implies $a_1 \leq r - 1$. □

Lemma B.3. *Let $\{R_1, \ldots, R_l, \ldots, R_r\}$ be the unique partition of the ordering $\{x_1, \ldots, x_N\}$ into inclusion-maximal runs of elements with the same condition labels. Then it holds that $r - 3 \leq U$.*

Proof. If $r \leq 3$, the lemma is trivial because the test statistic U of the Wilcoxon rank-sum test is non-negative. Hence, we can focus on the case $r \geq 4$. We now show that $r - 3 \leq U_0$. The inequality $r - 3 \leq U_1$ can be shown with an analogous argument, which then implies the desired result $r - 3 \leq \min\{U_0, U_1\} = U$.

Let $\mathcal{U}_0 = \{(x, x') \in \mathcal{X}_0 \times \mathcal{X}_1 \mid x < x'\}$ (note that we have $U_0 = |\mathcal{U}_0|$ by construction of $\mathcal{U}_0$). Moreover, for each $l \in [r]$, let b_l be R_l's boundary index — i. e., the index of the rightmost element in R_l — and let $\mathcal{B} = \{b_l \mid l \in \mathbb{N} \wedge 1 < l < r - 1\}$ be the set of all boundary indices except for the first and the last. By construction, we have $|\mathcal{B}| = r - 3$. For each boundary index $b_l \in \mathcal{B}$, we define $L_0(b_l)$ as the largest index of an element smaller or equal to x_{b_l} of class 0 and $R_1(b_l)$ as the smallest index of an element larger than x_{b_l} of class 1:

$$L_0(b_l) = \max\{i \in [N] \mid i \leq b_l \wedge x_i \in \mathcal{X}_0\} \tag{B.1}$$

$$R_1(b_l) = \min\{i \in [N] \mid i > b_l \wedge x_i \in \mathcal{X}_1\} \tag{B.2}$$

Since we have omitted the first and the last boundary from $\mathcal{B}$, $L_0(b_l)$ and $R_1(b_l)$ always exist. Moreover, $(x_{L_0(b_l)}, x_{R_1(b_l)}) \in \mathcal{U}_0$ holds by construction, which implies that $\phi(b_l) = (x_{L_0(b_l)}, x_{R_1(b_l)})$ defines a mapping from $\mathcal{B}$ to $\mathcal{U}_0$. This mapping is injective: Aiming for a contradiction, assume that there are boundaries $b_l, b_{l'} \in \mathcal{B}$ with $b_l < b_{l'}$ such that $\phi(b_l) = \phi(b_{l'})$. Pick an index $i \in [N]$ with $b_l < i \leq b_{l'}$ and consider the element x_i. Since $R_1(b_l) = R_1(b_{l'}) > b_{l'} \geq i$, we have $x_i \notin \mathcal{X}_1$. Since $L_0(b_{l'}) = L_0(b_l) \leq b_l < i$, we have $x_i \notin \mathcal{X}_0$. This contradicts $x_i \in \mathcal{X} = \mathcal{X}_0 \cup \mathcal{X}_1$. Hence, we have shown that $\phi : \mathcal{B} \to \mathcal{U}_0$ is injective, which implies $r - 3 = |\mathcal{B}| \leq |\mathcal{U}_0| = U_0$, as required. □

Combining the inequality $a_1 \leq r - 1$ from Lemma B.2 with the inequality $r - 3 < U$ from Lemma B.3 directly implies the statement of Theorem 1.

C Supplementary figures and tables

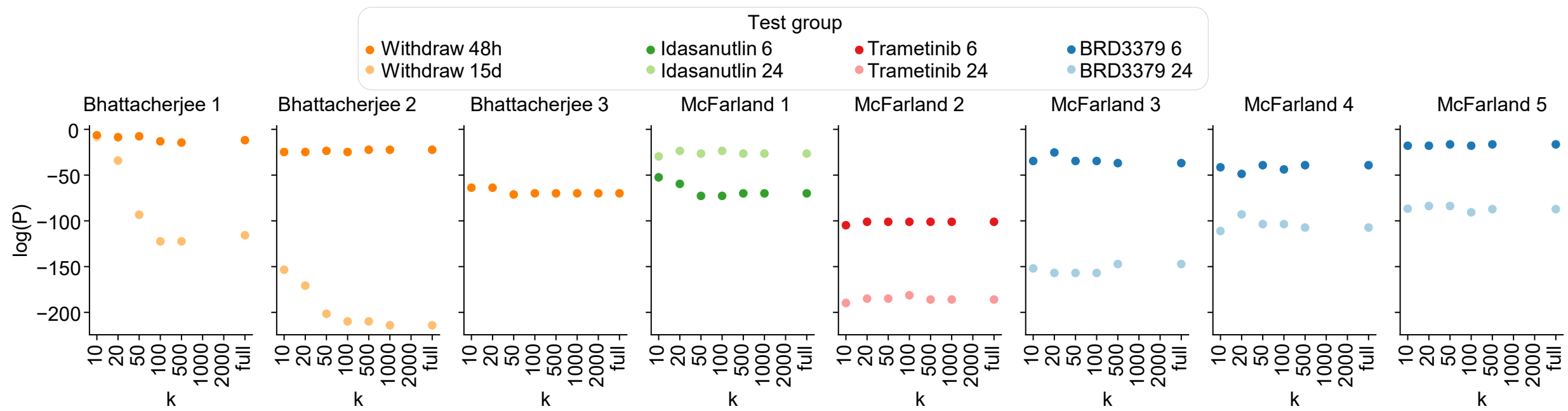

Fig. C.1: scXMatch P-values for testing each of the test groups against reference with different values of k. The P-values remain stable for all results with $k \geq 100$. Even for values of k far below the suggested default $k = 100$, the P-values only slightly vary from the ones retrieved in "full" mode, where scXMatch is run on a complete graph instead of a k-NN graph. Significance regarding $\alpha = 0.001$ is independent of k for all $k > 10$. The Norman and Schiebinger datasets were too large to run scXMatch in full graph mode and were therefore not included in this ablation.

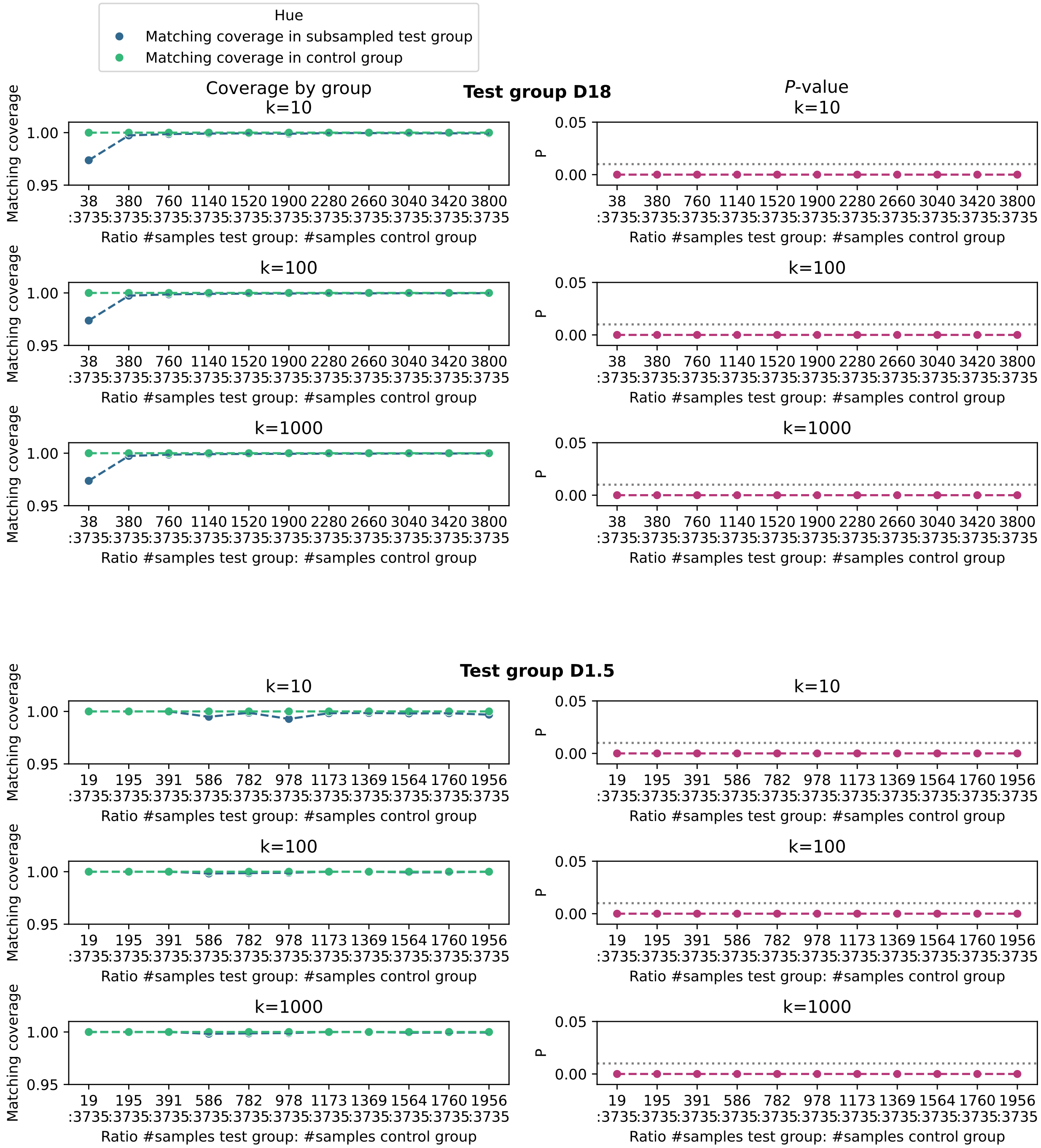

Fig. C.2: Results of the subsampling experiment on the Schiebinger dataset, using the control group (3735 cells) and the most strongly (day 18 with 3800 cells, top three rows) or the most weakly perturbed test group (day 1.5 with 1956 cells, bottom three rows). To assess scXMatch's robustness to class imbalance, we subsampled the test groups at ratios $r \in \{0.01, 0.1, 0.2, \ldots, 0.9\}$, left the control group untouched, and ran scXMatch on the increasingly imbalanced datasets for $k = \{10, 100, 1000\}$. Condition-stratified relative coverages are shown on the left, the right column shows the corresponding P-values. Both the relative coverages and the P-values remain remarkably stable: Even for $k = 10$ and a subsampling ratio of $r = 0.01$, only a single cell from the subsampled day 18 test group was not covered by the matching computed by scXMatch.

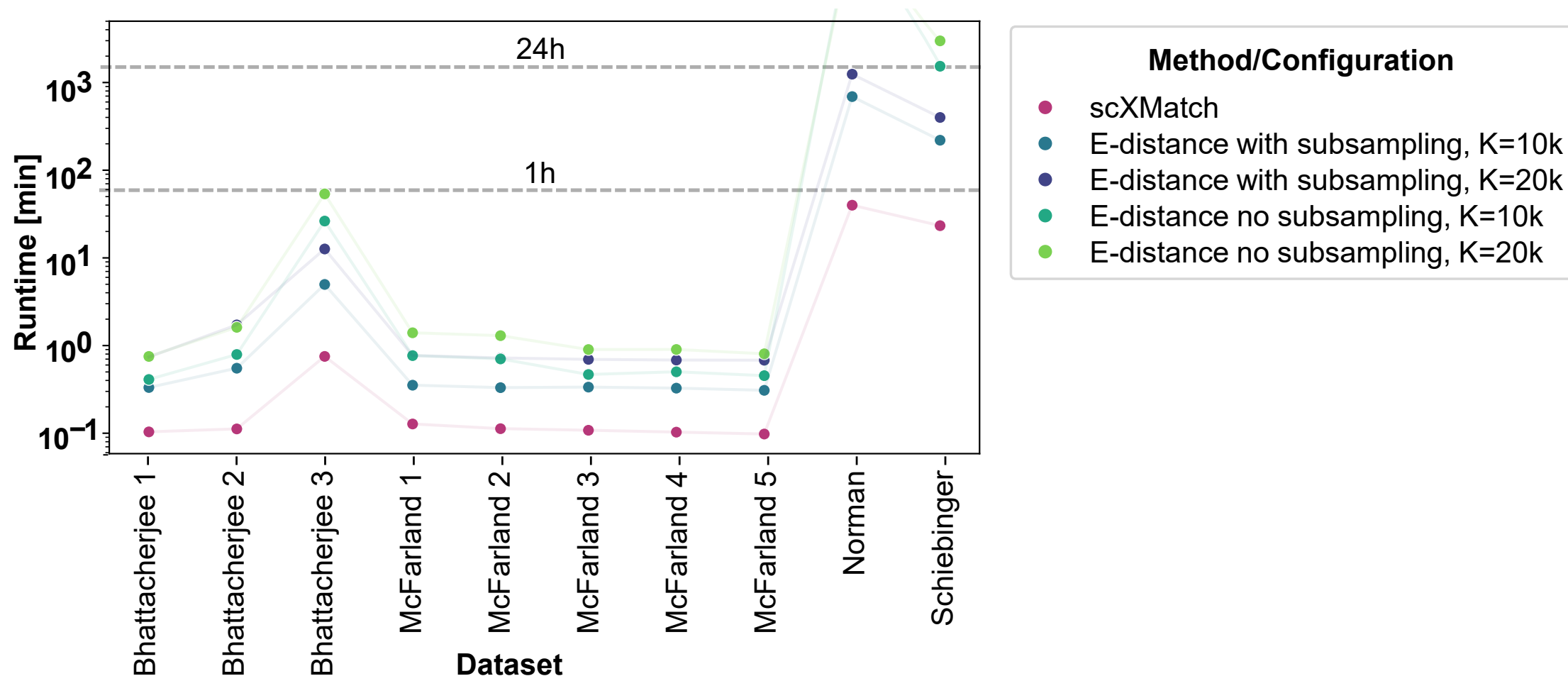

Fig. C.3: Direct comparison of the runtimes of scXMatch and the tested configurations (default and non-default) of the E-distance test. Note that due to infrastructural time constraints on our HPC system, the Norman and Schiebinger datasets had to be processed in chunks (perturbation groups had to be tested against reference separately). Still, the configurations without subsampling did not finish in under 24h each (or in under 48h in total) on the smallest possible chunks of the Norman dataset, hence the missing datapoints.

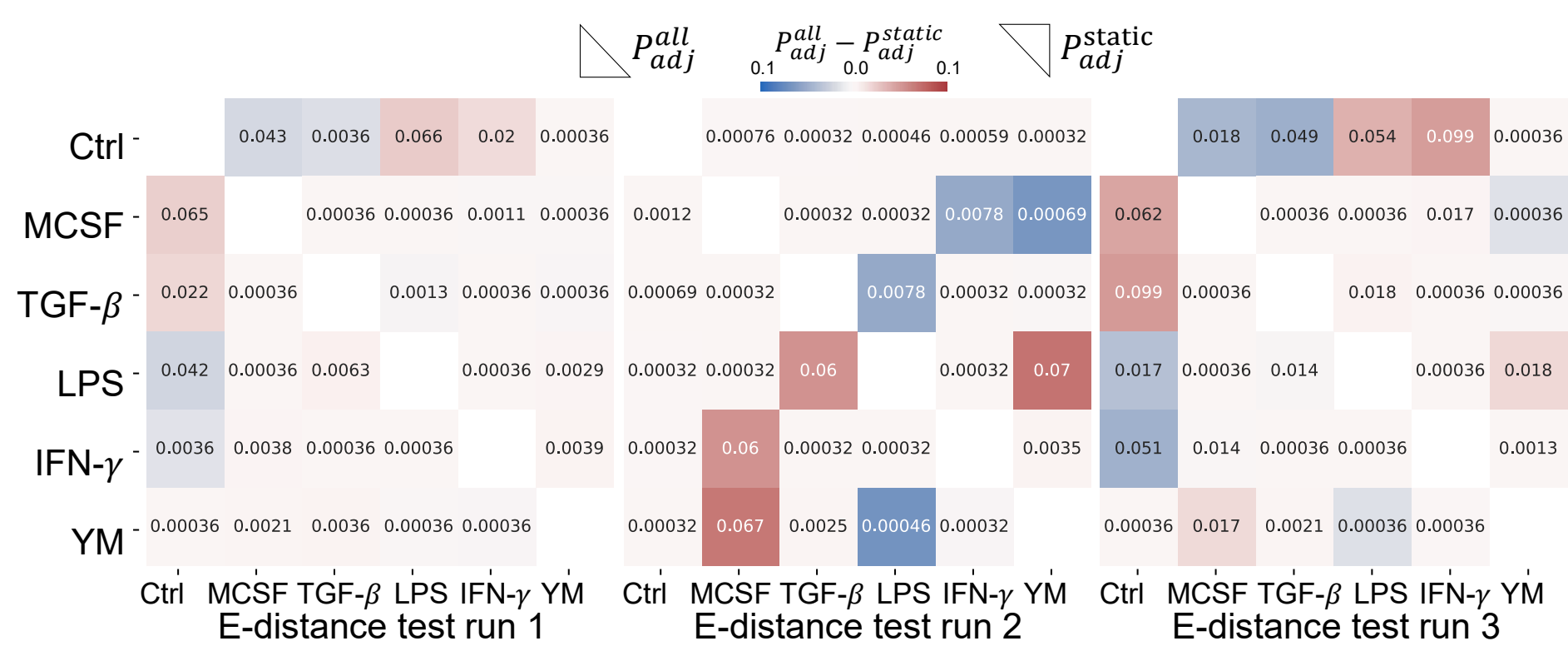

Fig. C.4: Repeated runs of the E-distance test on the same macrophage morphodynamics dataset. Due to the sample sizes in this dataset, non-deterministic nature of the random subsampling and the class label shuffling lead to substantially different results.

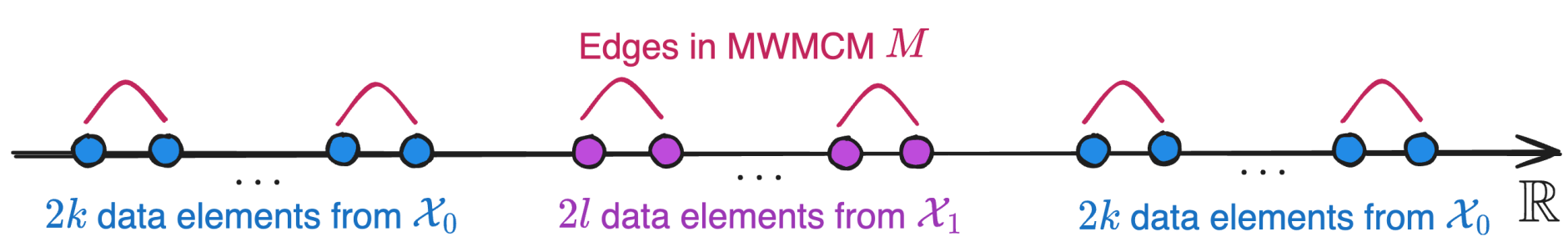

Fig. C.5: 1D example where scXMatch detects a distribution shift due to differences in spread that cannot be identified with the two-sided Wilcoxon rank-sum test. In the visualized arrangement, the $4k$ numbers from $\mathcal{X}_0 \subset \mathbb{R}$ (blue dots) and the $2l$ numbers from $\mathcal{X}_1 \subset \mathbb{R}$ (purple dots) have equal central tendencies and different spreads. The two-sided Wilcoxon rank-sum test does not detect this distribution shift but scXMatch does: Since there are no cross-matches in the MWMCM M computed by scXMatch (pink arcs), it holds that $a_1 = 0$. At the same time, the test statistic U of the Wilcoxon rank-sum test assumes the maximum possible value $U = 4kl = nm/2$: For each $x_j \in \mathcal{X}_1$, there are $2k$ elements $x_i \in \mathcal{X}_0$ with $x_i < x_j$ (implying $U_0 = 4kl$) and $2k$ elements $x_i \in \mathcal{X}_0$ with $x_i > x_j$ (implying $U_1 = 4kl$).

Table C.1: Resource usage of scXMatch for the tests on scRNA-seq datasets without subsampling.

**Dataset**	**Test group**	**Reference**	k	$N_{\mathbf{cells}}$	**Peak memory [GB]**	**Time [min]**	**Coverage [%]**
Bhattacherjee 1	withdraw 15d	maintenance	100	429	0.3	0.098	99.767
	withdraw 48h	maintenance	100	486	0.3	0.006	100.000
Bhattacherjee 2	withdraw 15d	maintenance	100	1231	0.0	0.016	99.919
	withdraw 48h	maintenance	100	955	0.0	0.096	99.895
Bhattacherjee 3	withdraw 15d	maintenance	100	6053	0.9	0.717	99.984
	withdraw 48h	maintenance	100	2952	0.9	0.035	100.000
McFarland 1	Idasanutlin 24	control	100	960	0.4	0.116	100.000
	Idasanutlin 6	control	100	935	0.4	0.011	99.893
McFarland 2	Trametinib 24	control	100	1020	0.3	0.103	100.000
	Trametinib 6	control	100	900	0.3	0.010	100.000
McFarland 3	BRD3379 24	control	100	733	0.0	0.101	99.864
	BRD3379 6	control	100	634	0.0	0.007	99.685
McFarland 4	BRD3379 24	control	100	731	0.4	0.095	99.863
	BRD3379 6	control	100	663	0.4	0.008	99.849
McFarland 5	BRD3379 24	control	100	636	0.0	0.091	100.000
	BRD3379 6	control	100	541	0.0	0.007	99.815
Norman	1	control	100	69570	4.6	25.865	99.997
	2	control	100	53520	4.6	14.004	99.993
Schiebinger	D1.5	control	100	5691	2.0	0.406	99.982
	D2	control	100	13405	2.0	1.830	99.993
	D2.5	control	100	10651	2.0	0.894	99.991
	D3	control	100	10556	2.0	0.910	99.981
	D3.5	control	100	11801	2.0	1.168	99.992
	D4	control	100	12697	2.0	1.092	99.992
	D4.5	control	100	10862	2.0	0.862	100.000
	D5	control	100	10962	2.0	0.825	100.000
	D5.5	control	100	10285	2.0	0.832	99.990
	D6	control	100	12157	2.0	1.011	99.992
	D6.5	control	100	6846	2.0	0.462	100.000
	D7	control	100	10242	2.0	0.750	100.000
	D7.5	control	100	8796	2.0	0.633	100.000
	D8	control	100	14823	2.0	1.525	99.993
	D8.5	control	100	7308	2.0	0.493	100.000
	D9	control	100	6814	2.0	0.475	100.000
	D9.5	control	100	6032	2.0	0.398	100.000
	D10	control	100	6154	2.0	0.433	100.000
	D10.5	control	100	5721	2.0	0.386	99.983
	D11	control	100	6347	2.0	0.449	99.984
	D11.5	control	100	5977	2.0	0.411	99.983
	D12	control	100	6164	2.0	0.421	100.000
	D12.5	control	100	5988	2.0	0.399	100.000
	D13	control	100	5881	2.0	0.395	99.983
	D13.5	control	100	6108	2.0	0.446	100.000
	D14	control	100	7493	2.0	0.594	99.987
	D14.5	control	100	6458	2.0	0.498	100.000
	D15	control	100	7658	2.0	0.617	100.000
	D15.5	control	100	8586	2.0	0.682	100.000
	D16	control	100	7177	2.0	0.576	99.986
	D16.5	control	100	8395	2.0	0.678	99.988
	D17	control	100	7413	2.0	0.558	99.987
	D17.5	control	100	7804	2.0	0.627	100.000
	D18	control	100	7535	2.0	0.559	99.987

References

1. Bhattacherjee, A., Djekidel, M.N., Chen, R., Chen, W., Tuesta, L.M., Zhang, Y.: Cell type-specific transcriptional programs in mouse prefrontal cortex during adolescence and addiction. Nat. Commun. **10**(1), 4169 (2019). https://doi.org/10.1038/s41467-019-12054-3
2. Dong, W., Moses, C., Li, K.: Efficient k-nearest neighbor graph construction for generic similarity measures. In: WWW 2011. pp. 577–586. ACM, New York, NY, USA (2011). https://doi.org/10.1145/1963405.1963487
3. Edmonds, J.: Maximum matching and a polyhedron with 0, 1-vertices. J. Nat. Bur. Stand. Sect. B **69**, 125–130 (1965)
4. Heumos, L., Ji, Y., May, L., Green, T.D., Peidli, S., Zhang, X., Wu, X., Ostner, J., Schumacher, A., Hrovatin, K., Müller, M., Chong, F., Sturm, G., Tejada, A., Dann, E., Dong, M., Pinto, G., Bahrami, M., Gold, I., Rybakov, S., Namsaraeva, A., Moinfar, A.A., Zheng, Z., Roellin, E., Mekki, I., Sander, C., Lotfollahi, M., Schiller, H.B., Theis, F.J.: Pertpy: an end-to-end framework for perturbation analysis. Nat. Methods **23**(2), 350–359 (2026). https://doi.org/10.1038/s41592-025-02909-7
5. Kim, M.C., Gate, R., Lee, D.S., Tolopko, A., Lu, A., Gordon, E., Shifrut, E., Garcia-Nieto, P.E., Marson, A., Ntranos, V., Ye, C.J.: Method of moments framework for differential expression analysis of single-cell RNA sequencing data. Cell **187**(22), 6393–6410.e16 (2024). https://doi.org/10.1016/j.cell.2024.09.044
6. Love, M.I., Huber, W., Anders, S.: Moderated estimation of fold change and dispersion for RNA-seq data with DESeq2. Genome Biol. **15**(12), 550 (2014). https://doi.org/10.1186/s13059-014-0550-8
7. McFarland, J.M., Paolella, B.R., Warren, A., Geiger-Schuller, K., Shibue, T., Rothberg, M., Kuksenko, O., Colgan, W.N., Jones, A., Chambers, E., Dionne, D., Bender, S., Wolpin, B.M., Ghandi, M., Tirosh, I., Rozenblatt-Rosen, O., Roth, J.A., Golub, T.R., Regev, A., Aguirre, A.J., Vazquez, F., Tsherniak, A.: Multiplexed single-cell transcriptional response profiling to define cancer vulnerabilities and therapeutic mechanism of action. Nat. Commun. **11**(1), 4296 (2020). https://doi.org/10.1038/s41467-020-17440-w
8. Mimitou, E.P., Lareau, C.A., Chen, K.Y., Zorzetto-Fernandes, A.L., Hao, Y., Takeshima, Y., Luo, W., Huang, T.S., Yeung, B.Z., Papalexi, E., Thakore, P.I., Kibayashi, T., Wing, J.B., Hata, M., Satija, R., Nazor, K.L., Sakaguchi, S., Ludwig, L.S., Sankaran, V.G., Regev, A., Smibert, P.: Scalable, multimodal profiling of chromatin accessibility, gene expression and protein levels in single cells. Nat. Biotechnol. **39**(10), 1246–1258 (2021). https://doi.org/10.1038/s41587-021-00927-2
9. Muzellec, B., Teleńczuk, M., Cabeli, V., Andreux, M.: PyDESeq2: a python package for bulk RNA-seq differential expression analysis. Bioinformatics **39**(9) (2023). https://doi.org/10.1093/bioinformatics/btad547
10. Norman, T.M., Horlbeck, M.A., Replogle, J.M., Ge, A.Y., Xu, A., Jost, M., Gilbert, L.A., Weissman, J.S.: Exploring genetic interaction manifolds constructed from rich single-cell phenotypes. Science **365**(6455), 786–793 (2019). https://doi.org/10.1126/science.aax4438
11. Okuta, R., Unno, Y., Nishino, D., Hido, S., Loomis, C.: CuPy: A NumPy-compatible library for NVIDIA GPU calculations. In: Proceedings of Workshop on Machine Learning Systems (LearningSys) in The Thirty-first Annual Conference on Neural Information Processing Systems (NIPS) (2017)
12. Peidli, S., Green, T.D., Shen, C., Gross, T., Min, J., Garda, S., Yuan, B., Schumacher, L.J., Taylor-King, J.P., Marks, D.S., Luna, A., Blüthgen, N., Sander, C.: scPerturb: harmonized single-cell perturbation data. Nat. Methods **21**(3), 531–540 (2024). https://doi.org/10.1038/s41592-023-02144-y
13. Peixoto, T.P.: The graph-tool python library. figshare (2014). https://doi.org/10.6084/m9.figshare.1164194
14. Robinson, M.D., McCarthy, D.J., Smyth, G.K.: edgeR: a bioconductor package for differential expression analysis of digital gene expression data. Bioinformatics **26**(1), 139–140 (2010). https://doi.org/10.1093/bioinformatics/btp616
15. Rosenbaum, P.R.: An exact distribution-free test comparing two multivariate distributions based on adjacency. J. R. Stat. Soc. Series B Stat. Methodol. **67**(4), 515–530 (2005). https://doi.org/10.1111/j.1467-9868.2005.00513.x
16. Schiebinger, G., Shu, J., Tabaka, M., Cleary, B., Subramanian, V., Solomon, A., Gould, J., Liu, S., Lin, S., Berube, P., Lee, L., Chen, J., Brumbaugh, J., Rigollet, P., Hochedlinger, K., Jaenisch, R., Regev, A., Lander, E.S.: Optimal-transport analysis of single-cell gene expression identifies developmental trajectories in reprogramming. Cell **176**(4), 928–943.e22 (2019). https://doi.org/10.1016/j.cell.2019.01.006
17. Schnitzerlein, M., Greto, E., Wegner, A., Möller, A., Aust, O., Ben Brahim, O., Blumenthal, D.B., Zaburdaev, V., Uderhardt, S.: Cellular morphodynamics as quantifiers for functional states of resident tissue macrophages in vivo. PLoS Comput. Biol. **21**(5), e1011859 (2025). https://doi.org/10.1371/journal.pcbi.1011859
18. Squair, J.W., Gautier, M., Kathe, C., Anderson, M.A., James, N.D., Hutson, T.H., Hudelle, R., Qaiser, T., Matson, K.J.E., Barraud, Q., Levine, A.J., La Manno, G., Skinnider, M.A., Courtine, G.: Confronting false discoveries in single-cell differential expression. Nat. Commun. **12**(1), 5692 (2021). https://doi.org/10.1038/s41467-021-25960-2
19. Virshup, I., Bredikhin, D., Heumos, L., Palla, G., Sturm, G., Gayoso, A., Kats, I., Koutrouli, M., Scverse Community, Berger, B., Pe'er, D., Regev, A., Teichmann, S.A., Finotello, F., Wolf, F.A., Yosef, N., Stegle, O., Theis,

F.J.: The scverse project provides a computational ecosystem for single-cell omics data analysis. Nat. Biotechnol. **41**(5), 604–606 (2023). https://doi.org/10.1038/s41587-023-01733-8

20. Virshup, I., Rybakov, S., Theis, F.J., Angerer, P., Wolf, F.A.: anndata: Access and store annotated data matrices. J. Open Source Softw. **9**(101), 4371 (2024). https://doi.org/10.21105/joss.04371
21. Virtanen, P., Gommers, R., Oliphant, T.E., Haberland, M., Reddy, T., Cournapeau, D., Burovski, E., Peterson, P., Weckesser, W., Bright, J., van der Walt, S.J., Brett, M., Wilson, J., Millman, K.J., Mayorov, N., Nelson, A.R.J., Jones, E., Kern, R., Larson, E., Carey, C.J., Polat, İ., Feng, Y., Moore, E.W., VanderPlas, J., Laxalde, D., Perktold, J., Cimrman, R., Henriksen, I., Quintero, E.A., Harris, C.R., Archibald, A.M., Ribeiro, A.H., Pedregosa, F., van Mulbregt, P., SciPy 1.0 Contributors: SciPy 1.0: fundamental algorithms for scientific computing in Python. Nat. Methods **17**(3), 261–272 (2020). https://doi.org/10.1038/s41592-019-0686-2
22. Wilcoxon, F.: Individual comparisons by ranking methods. In: Springer Series in Statistics, pp. 196–202. Springer New York, New York, NY (1992). https://doi.org/10.1007/978-1-4612-4380-9_16
23. Wolf, F.A., Angerer, P., Theis, F.J.: SCANPY: large-scale single-cell gene expression data analysis. Genome Biol. **19**(1), 15 (2018). https://doi.org/10.1186/s13059-017-1382-0
24. Zappia, L., Phipson, B., Oshlack, A.: Splatter: simulation of single-cell RNA sequencing data. Genome Biol. **18**(1), 174 (2017). https://doi.org/10.1186/s13059-017-1305-0
